# Regional variations in hepatocellular carcinoma incidence, routes to diagnosis, treatment and survival in England

**DOI:** 10.1038/s41416-021-01509-4

**Published:** 2021-11-26

**Authors:** Anya Burton, Vinay K. Balachandrakumar, Robert J. Driver, Daniela Tataru, Lizz Paley, Aileen Marshall, Graeme Alexander, Ian A. Rowe, HCC-UK/BASL/NCRAS Partnership, Daniel H. Palmer, Tim J. S. Cross

**Affiliations:** 1 HCC-UK/British Association for the Study of the Liver, Lichfield, UK; 2 National Cancer Registration and Analysis Service (NCRAS), Public Health England, London, UK; 3 Department of Molecular and Clinical Cancer Medicine, Institute of Translational Medicine, University of Liverpool, Liverpool, UK; 4 Leeds Institute for Medical Research at St. James’s, University of Leeds, Leeds, UK; 5 Sheila Sherlock Liver Centre, The Royal Free Hospital, London, UK; 6 Institute for Liver and Digestive Health, Royal Free Hospital Pond St, Hampstead, London, UK; 7 Liverpool Experimental Cancer Medicine Centre, University of Liverpool, Liverpool, UK

**Keywords:** Cancer epidemiology, Hepatocellular carcinoma

## Abstract

**Background:**

Hepatocellular carcinoma (HCC) incidence, management and survival across England were examined to determine if geographical inequalities exist.

**Method:**

15,468 HCC cases diagnosed 2010–2016 were included. Age-standardised incidence rates, net survival and proportions receiving potentially curative treatment and presenting through each route to diagnosis adjusted for age at diagnosis, sex and area-based deprivation quintile, were calculated overall and by Cancer Alliance.

**Results:**

HCC incidence rates increased in men from 6.2 per 100,000 in 2010 to 8.8 in 2016, and in women from 1.5 to 2.2. The highest incidence rates, found in parts of the North of England and London, were nearly double the lowest. The adjusted proportion presenting as an emergency ranged 27–41% across Cancer Alliances. Odds increased with increasing deprivation quintile and age. Only one in five patients received potentially curative treatment (range 15–28%) and odds decreased with increasing deprivation and age. One-year survival in 2013–2016 ranged 38–53%.

**Conclusion:**

This population-based, nationwide analysis demonstrates clear differences in HCC incidence, management and survival across England. It highlights socioeconomic-associated variation and the need for improvement in early diagnosis and curative treatment of HCC. This research should assist policymakers, service providers and clinicians to identify regions where additional training, services and resources would be best directed.

## Background

The world age-standardised incidence rate of primary liver cancer varies widely globally, from 94 per 100,000 in Mongolia to 1.1 per 100,000 in Morocco [1]. There is also geographical variation in the rate of change in incidence rates, with rates decreasing in recent decades in countries such as Japan and Poland, and increasing in countries such as the Netherlands, the USA and the UK [2]. This may reflect different aetiological factors, development of new therapies, and global health initiatives. Hepatocellular carcinoma (HCC) is the most common type of primary liver cancer and predominately occurs in patients with liver cirrhosis [3]. In England, the main causes of cirrhosis and consequently HCC are hepatitis C, alcohol-related liver disease, obesity and hepatitis B [2, 4, 5]. HCC survival is poor, with less than 15% of patients alive 5 years after diagnosis in the UK [6]. Prognosis is dependent on many factors including cancer stage at diagnosis, underlying liver function, performance status and access to treatments [7]. Liver surveillance via ultrasound is recommended for adults with cirrhosis [8].

As well as variation in HCC across countries, there is variation within countries, as previously demonstrated in the USA and France and across the UK nations [6, 9, 10]. Geographic variation in aspects of liver disease within England were explored in the 2nd Atlas of Variation, 2017 [5]. The highest chronic liver disease mortality rates were seen in clinical commissioning groups in the North East and North West of England, where rates were two to three times higher than the national average. An outcome from this report was more detailed work was needed to understand the changing pattern of HCC and how it affects patients and clinical services in different regions. Exploring geographical variations in risk, management and survival of HCC can identify areas where targeted interventions could be used to reduce incidence and facilitate early diagnosis and treatment of HCC, leading to improved patient survival.

Building on previous work exploring variation in the epidemiology of liver cancer across the UK [6], here we aim to look specifically at differences in HCC across England, particularly in the causes and management. The specific aims of the current study were to determine if there was regional variation across the 19 English Cancer Alliances in the following areas: (1) incidence and change in incidence of HCC over the study period, (2) cause of underlying liver disease, (3) route to diagnosis of HCC; (4) proportion receiving potentially curative treatments; (5) survival.

## Methods

Data on all patients resident in England diagnosed with HCC (defined as ICD10 C22.0 and the ICDO2 morphology code M8170) between 1st January 2010 and 31^st^ December 2016 were extracted from the National Cancer Registration Dataset [11]. 60% of diagnoses were based on clinical investigations such as imaging (as recommended for cirrhotic patients in the EASL Clinical Practice Guidelines [12]), 35% were based on pathology, and the remaining 5% were death certificate only or the basis of diagnosis was unknown. The English cancer registry includes all cases of cancer diagnosed and treated in the National Health Service (NHS) in England, which funds 98–99% of all hospital activity [13], and also some treated privately [11]. The National Cancer Registration Dataset is outlined in detail in Henson et al. [11], but data feeds are extensive and include multidisciplinary teams meetings, hospital activity records, patient administration systems in hospitals, pathology reports, molecular test reports and death certification from the Office for National Statistics. The proportion of cancers identified through death certification alone, a marker of registry quality, is less than 1%. This indicates that the large majority of data relevant to a cancer diagnosis is being captured and hence very high population completeness of the cancer registry [11].

Linked Hospital Episodes Statistics Admitted Patient Care (HES APC) captures all inpatient NHS hospital admissions in England and 98.9% of HCC patients had at least one HES inpatient record. The cohort had vital status follow up data up to 31st December 2017. The extracted data included: date of diagnosis, age at diagnosis, sex, route to diagnosis, socioeconomic deprivation quintile, ethnicity, vital status and vital status date. The measure of socioeconomic deprivation used was based on the income domain score of the English Indices of Multiple Deprivation (IMD) 2010 [14] which measures relative levels of deprivation in small areas of England called Lower layer Super Output Areas (LSOAs). LSOAs, together with their mid-year population estimates, were sorted according to the raw scores of the income domain. LSOAs were then grouped into quintiles (1 being the least deprived, 5 being the most deprived) so that each contains approximately 20% of the population of England. Patients were assigned to an LSOA and subsequently a socioeconomic deprivation quintile based on their postcode of residence at the time of diagnosis. Patients aged under 20 were excluded (*n* = 22) due to rare causes of HCC in young patients. To assess geographical variation, all patients were assigned to one of the 19 Cancer Alliances based on their postcode of residence at the time of diagnosis. Cancer Alliances are partnerships of health and social care organisations with designated population catchment areas covering the whole of England which were designed to drive local change in the quality of cancer services to improve cancer outcomes and patient experience [15]. There are regular changes to the structure of the Cancer Alliances; here we present the results using the 2017 structure (Supplementary Fig. 1).

Mid-year population estimates were obtained from the Office for National Statistics for each of the years 2010–2016 and age-standardised incidence rates (ASRs) were calculated using the European Standard Population 2013 [16].

A route to diagnosis (RTD) was assigned to each cancer using a well-documented algorithm [17]. The algorithm takes the date of cancer diagnosis, as defined by the UK and Ireland Association of Cancer Registries using European Network of Cancer Registries rules, as the starting point. Routine data immediately prior to this date are examined and a series of rules are applied to classify the RTD for each case. The RTD categories are emergency presentation, general practice (GP) referral, Two Week Wait (TWW, urgent GP Referral with a suspicion of cancer), inpatient elective, other-outpatient, death certification only (DCO), and unknown.

Treatment data were extracted from the National Cancer Registration Dataset and the HES APC dataset. Any treatment from 60 days prior to diagnosis until death or end of treatment data follow up (March 2018) was included. Flexibility around the date of diagnosis was allowed as HCC may be diagnosed clinically and treated prior to the official registry date of incidence, which can be redefined once histological tissue is received [18]. The most definitive potentially curative treatment a patient received during this time point, irrespective of other treatments received, was captured based on the following hierarchy: liver transplant > liver resection > radiofrequency or microwave ablation > irreversible electroporation > percutaneous ethanol injection. The median time from diagnosis to potentially curative treatment was 61 days. Underlying causes of primary liver disease (PLD) were identified from diagnostic codes recorded in HES APC records occurring 5 years before to 1 year after HCC diagnosis. Codes indicative of chronic hepatitis C or B (HCV and HBV), primary biliary cholangitis (PBC), autoimmune hepatitis (AIH), haemochromatosis, alcoholic liver disease (ALD), or non-alcoholic fatty liver disease (NAFLD) were included. NAFLD was defined as fatty (change of) liver, not elsewhere classified, or by the presence of cirrhosis combined with obesity or diabetes without the presence of any other PLD. A hierarchy was applied based on relative risk associated with each aetiology (HCV > HBV > PBC > AIH > haemochromatosis > ALD > NAFLD) so that one ‘primary’ aetiology was assigned per patient. Any patients without evidence of a PLD were assigned to the ‘Other/Unknown’ category. Due to small numbers, AIH and PBC were combined into one category and haemochromatosis was added to the other/unknown category for this analysis.

### Patient and public involvement statement

No patients were involved in forming the research question or selecting the outcome measures, nor were they involved in developing the study design. No patients were asked to advise on interpretation or writing up of results. Results will be shared through patient charities, regionally and nationally, including the British Liver Trust and on the NCRAS and British Association for the Study of the Liver websites.

### Statistical analysis

The distribution of demographic factors among HCC cases by Cancer Alliance were described using means and standard deviation or absolute numbers and percentages. Logistic regression models were used to explore differences in the proportions of patients presenting via each route versus all other routes by Cancer Alliance, unadjusted and adjusted for demographic variables age (as a continuous variable), sex and deprivation quintile (as a continuous variable). Similar logistic regression models were used to explore differences in proportions of patients receiving potentially curative treatments by Cancer Alliance. Logistic regression models were also used to explore variation in primary liver disease aetiology by Cancer Alliance, unadjusted and adjusted for age, sex, deprivation quintile and ethnicity. Data on age, sex and deprivation quintile were complete. Unknown ethnicity (4.8% of patients) was included as a separate category. Odds ratios were converted to proportions and the p-value for difference between the 19 Cancer Alliances calculated using the likelihood ratio test.

One-and two-year net survival was estimated and the Brenner method of age-adjustment applied [19]. Due to sparse data in some groups, survival by year could not be estimated. Instead, four-year cohorts were used to assess trends (2010–2013 to 2013–2016). Survival estimates were suppressed if there were no deaths or data in at least one age band, less than 10 in a group, the standard error was greater than 0.2 and/or the follow up time was insufficient in a cohort.

Results were displayed in choropleth maps (in quintiles) and in forest plots to show differences across regions.

The summary metrics for each Cancer Alliance were compared using pairwise correlation coefficients.

Statistical analysis was performed using Stata version 15.1 (StataCorp, College Station, TX).

This study was approved within the National Cancer Registration and Analysis Service, Public Health England, under regulation 2 of the Health Service (Control of Patient Information) Regulations 2002.

## Results

### Descriptive

Between 2010 and 2016, a total of 15,468 patients were diagnosed with HCC. Mean age at HCC diagnosis was 69.2 years, which varied by Cancer Alliance (65.2 years in North Central and North East London to 71.5 years in North East and Cumbria) (Table 1). 77.8% of cases were in men, which also varied, from 75.4% in Cheshire and Merseyside to 80.2% in North Central and North East London. Overall, 15.6% of cases were from the least and 24.8% of cases were from the most socioeconomically deprived areas. This varied by Cancer Alliance, largely reflecting differences in their sociodemographic structures. For example, in North Central and North East London, 50% of the general population and 42.2% of HCC cases are in the most deprived quintile. Conversely in Thames Valley, just 5% of the general population and 6.8% of the HCC cases were in the most deprived quintile.

**Table 1:** Description of cases by Cancer Alliance.

	Cases	Age		Sex				Deprivation Quintile*									
				Male		Female		1 (least deprived)		2		3		4		5 (most deprived)	
	N	Mean	SD	N	%	N	%	N	%	N	%	N	%	N	%	N	%
England Overall	15,468	69.2	(11.8)	12,034	(77.8%)	3,434	(22.2%)	2,407	(15.6%)	2,895	(18.7%)	3,074	(19.9%)	3,253	(21.0%)	3,839	(24.8%)
*By Cancer Alliance*																	
West Yorkshire	844	69.6	(12.1)	652	(77.3%)	192	(22.7%)	126	(14.9%)	165	(19.5%)	138	(16.4%)	162	(19.2%)	253	(30.0%)
Humber, Coast and Vale	375	70.9	(10.6)	295	(78.7%)	80	(21.3%)	70	(18.7%)	79	(21.1%)	89	(23.7%)	61	(16.3%)	76	(20.3%)
Cheshire and Merseyside	963	68.6	(11.3)	726	(75.4%)	237	(24.6%)	123	(12.8%)	146	(15.2%)	138	(14.3%)	161	(16.7%)	395	(41.0%)
South Yorkshire, Bassetlaw, North Derbyshire and Hardwick	667	70.4	(11.6)	508	(76.2%)	159	(23.8%)	83	(12.4%)	110	(16.5%)	121	(18.1%)	138	(20.7%)	215	(32.2%)
West Midlands	1,596	69.7	(11.7)	1,230	(77.1%)	366	(22.9%)	198	(12.4%)	286	(17.9%)	297	(18.6%)	334	(20.9%)	481	(30.1%)
East Midlands	960	69.0	(11.6)	743	(77.4%)	217	(22.6%)	159	(16.6%)	191	(19.9%)	212	(22.1%)	197	(20.5%)	201	(20.9%)
East of England	1,610	69.1	(11.8)	1,250	(77.6%)	360	(22.4%)	293	(18.2%)	398	(24.7%)	433	(26.9%)	295	(18.3%)	191	(11.9%)
South East London	417	65.9	(13.4)	321	(77.0%)	96	(23.0%)	38	(9.1%)	40	(9.6%)	81	(19.4%)	124	(29.7%)	134	(32.1%)
Kent and Medway	397	69.8	(10.8)	303	(76.3%)	94	(23.7%)	64	(16.1%)	83	(20.9%)	99	(24.9%)	83	(20.9%)	68	(17.1%)
Surrey and Sussex	749	71.2	(11.3)	599	(80.0%)	150	(20.0%)	219	(29.2%)	191	(25.5%)	161	(21.5%)	118	(15.8%)	60	(8.0%)
Thames Valley	497	68.2	(12.4)	387	(77.9%)	110	(22.1%)	183	(36.8%)	110	(22.1%)	96	(19.3%)	74	(14.9%)	34	(6.8%)
Peninsula	510	70.3	(11.5)	403	(79.0%)	107	(21.0%)	46	(9.0%)	101	(19.8%)	168	(32.9%)	125	(24.5%)	70	(13.7%)
Somerset, Wiltshire, Avon and Gloucestershire	729	70.2	(11.2)	563	(77.2%)	166	(22.8%)	155	(21.3%)	200	(27.4%)	158	(21.7%)	132	(18.1%)	84	(11.5%)
Wessex	747	70.7	(11.0)	594	(79.5%)	153	(20.5%)	179	(24.0%)	174	(23.3%)	156	(20.9%)	169	(22.6%)	69	(9.2%)
North East and Cumbria	1,109	71.5	(10.5)	874	(78.8%)	235	(21.2%)	156	(14.1%)	155	(14.0%)	145	(13.1%)	262	(23.6%)	391	(35.3%)
Lancashire and South Cumbria	568	69.2	(12.2)	444	(78.2%)	124	(21.8%)	65	(11.4%)	121	(21.3%)	104	(18.3%)	99	(17.4%)	179	(31.5%)
Greater Manchester	1,027	68.5	(12.1)	811	(79.0%)	216	(21.0%)	123	(12.0%)	150	(14.6%)	157	(15.3%)	202	(19.7%)	395	(38.5%)
North Central and North East London	834	65.2	(13.2)	669	(80.2%)	165	(19.8%)	30	(3.6%)	67	(8.0%)	108	(12.9%)	275	(33.0%)	354	(42.4%)
North West and South West London	869	67.1	(12.0)	662	(76.2%)	207	(23.8%)	97	(11.2%)	128	(14.7%)	213	(24.5%)	242	(27.8%)	189	(21.7%)

*Based on the income domain of the Index of Multiple Deprivation.

### Incidence

**Fig. 1:**
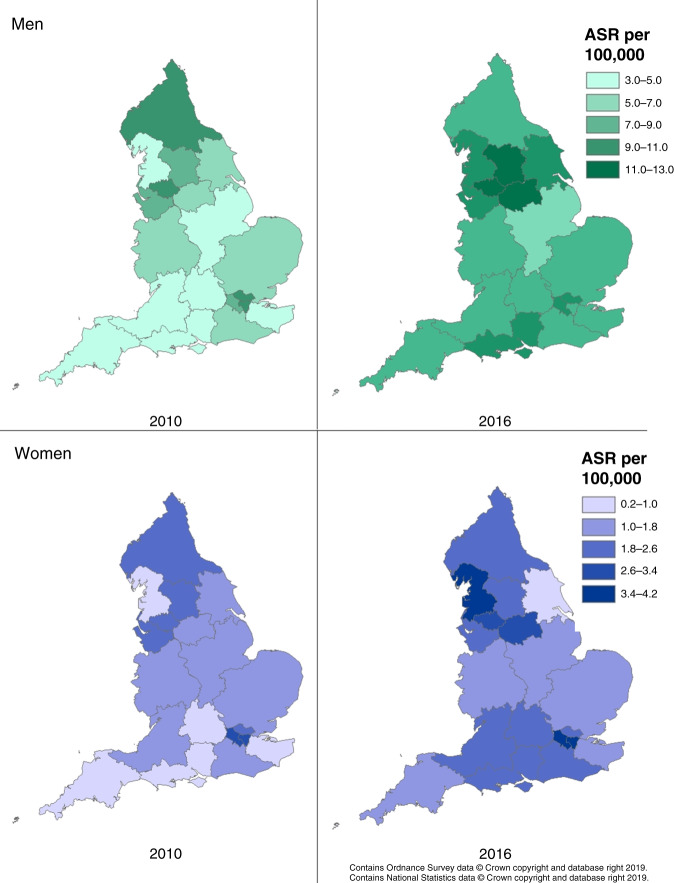
HCC incidence by Cancer Alliance. By year: 2010 on the left and 2016 on the right. By Sex: Men on the first row, women on the second row.

Incidence rates varied greatly by sex and therefore are displayed separately for men and women. Between 2010 and 2016, there was an increase in the age-standardised incidence rate (ASR) of HCC in both men and women nationally; the ASR increased from 6.2 (95% confidence interval (CI) 5.9–6.5) per 100,000 to 8.8 (95%CI 8.4–9.2) in men and from 1.5 (95%CI 1.3–1.6) to 2.2 (95%CI 2.0–2.4) per 100,000 in women (Supplementary Table 1). Most Cancer Alliances saw an increase, though in some Cancer Alliances incidence rates remained relatively stable, for example in South East London. By 2016, the highest incidence rates were in the North West of England and London; in men, Greater Manchester had the highest ASR at 12.1 (95%CI 10.1–14.4) and in women, North West and South West London at 3.9 (95%CI 2.9–5.1) per 100,000 (Fig. 1). The lowest ASR was seen in East Midlands among men 6.8 (95%CI 5.6–8.1) and in Humber, Coast and Vale among women 0.9 (95%CI 0.4–1.9) per 100,000.

### Underlying cause of primary liver disease (pld)

Across HCC cases, ALD was the most common PLD identified (21.0%), followed by viral hepatitis (17.2%), NAFLD (16.1%) and then AIH and PBC (3.1%). In the remaining 42.7%, underlying PLD did not fall into one of these categories, could not be identified, or there was no PLD. Differences in the proportion by Cancer Alliance are given in Table 2, unadjusted and adjusted for age, sex, deprivation quintile and ethnicity. The proportion with ALD-associated HCC ranged from 15.2 to 27.2% across Cancer Alliances prior to adjustment (*p* for difference between Cancer Alliances <0.001), which was somewhat attenuated following adjustment to 14.8–23.7% (*p* < 0.001). It was most common in Cheshire and Merseyside and North East and Cumbria. Geographical variation in viral hepatitis was wide (6.9–41.6% before adjustment) and adjustment reduced this substantially (to 5.8–22.0%) as there were strong associations with age (odds 8% lower per year increase in age at diagnosis), sex (odds 23% lower in women), deprivation (odds 20% higher per quintile increase in deprivation) and ethnicity (odds over three times higher in people of all other ethnicities compared to white people). It was most common in the London Cancer Alliances (18–22%, after adjustment) and least in Humber, Coast and Vale, and North East and Cumbria (6.4% and 5.8%, respectively) (Supplementary Fig. 2). There was less geographical variation in the proportion with NAFLD-associated HCC (11.6–20.7%, *p* < 0.001 pre-adjustment and 12.4–19.1%, *p* = 0.001 post-adjustment), and odds were 11% higher in women, increased with increasing age, were highest in white and South Asian and lowest in black people. The proportion of cases with NAFLD-associated HCC was lowest in North West and South West London and highest in North East and Cumbria, Peninsula and Wessex. The proportion with PBC or AIH ranged from 1.6 to 4.9% across Cancer Alliances prior to adjustment (*p* = 0.008), and from 0.8 to 2.7% after adjustment (*p* = 0.023). The proportion was lowest in Thames Valley and highest in the East Midlands, although the confidence intervals were wide. The proportion in the ‘Other/Unknown’ group varied widely across Cancer Alliances from 28.2 to 53.8% (*p* < 0.001), which was only slightly attenuated following adjustment (to 31.9–51.0%, p < 0.001). The lowest proportion was in North Central and North East London and the highest was in the Humber, Coast and Vale, and South Yorkshire, Bassetlaw, North Derbyshire and Hardwick Cancer Alliances.

**Table 2:** Proportion with each primary liver disease by Cancer Alliance.

	ALD			Viral hepatitis			NAFLD			AIH and PBC			Other		
	Percent	95% CI	*p*	Percent	95% CI	*p*	Percent	95% CI	*p*	Percent	95% CI	*p*	Percent	95% CI	*p*
England Overall	21.0%			17.2%			16.1%			3.1%			42.7%		
*By Cancer Alliance, unadjusted*															
West Yorkshire	21.9%	(19.2–24.8%)		15.9%	(13.6–18.5%)		15.2%	(12.9–17.7%)		2.6%	(1.7–3.9%)		44.5%	(41.2–47.9%)	
Humber, Coast & Vale	19.4%	(15.8–23.7%)		7.3%	(5.1–10.4%)		17.3%	(13.8–21.5%)		2.1%	(1.1–4.1%)		53.8%	(48.8–58.8%)	
Cheshire and Merseyside	27.2%	(24.5–30.1%)		15.1%	(13.0–17.5%)		14.8%	(12.7–17.1%)		4.3%	(3.2–5.8%)		38.6%	(35.6–41.7%)	
South Yorkshire, Bassetlaw, North Derbyshire & Hardwick	17.1%	(14.4–20.1%)		11.8%	(9.6–14.4%)		15.7%	(13.2–18.7%)		3.5%	(2.4–5.2%)		51.9%	(48.2–55.6%)	
West Midlands	20.3%	(18.4–22.3%)		14.0%	(12.4–15.8%)		16.2%	(14.5–18.0%)		3.2%	(2.5–4.2%)		46.3%	(43.9–48.8%)	
East Midlands	18.0%	(15.7–20.6%)		14.2%	(12.1–16.5%)		17.0%	(14.8–19.5%)		4.9%	(3.7–6.4%)		45.9%	(42.8–49.1%)	
East of England	18.4%	(16.6–20.4%)		17.1%	(15.4–19.1%)		17.8%	(16.0–19.7%)		3.6%	(2.8–4.6%)		43.1%	(40.7–45.5%)	
South East London	16.4%	(13.2–20.2%)		34.0%	(29.7–38.6%)		11.6%	(8.9–14.9%)		2.5%	(1.4–4.5%)		35.4%	(31.0–40.0%)	
Kent & Medway	25.1%	(21.1–29.6%)		13.7%	(10.7–17.4%)		16.7%	(13.3–20.6%)		4.2%	(2.6–6.7%)		40.3%	(35.6–45.2%)	
Surrey & Sussex	21.5%	(18.7–24.6%)		15.7%	(13.3–18.5%)		16.0%	(13.6–18.8%)		2.1%	(1.3–3.4%)		44.7%	(41.2–48.2%)	
Thames Valley	22.7%	(19.3–26.6%)		18.6%	(15.4–22.2%)		13.8%	(11.1–17.1%)		1.6%	(0.8–3.1%)		43.3%	(39.0–47.6%)	
Peninsula	23.4%	(19.9–27.2%)		14.1%	(11.4–17.4%)		19.5%	(16.3–23.1%)		3.1%	(1.9–5.0%)		40.0%	(35.8–44.2%)	
Somerset, Wiltshire, Avon & Gloucestershire	20.5%	(17.7–23.5%)		13.5%	(11.2–16.1%)		18.3%	(15.7–21.2%)		2.7%	(1.7–4.1%)		45.1%	(41.5–48.7%)	
Wessex	23.0%	(20.1–26.1%)		11.9%	(9.8–14.4%)		19.8%	(17.1–22.8%)		2.9%	(1.9–4.4%)		42.4%	(38.9–46.0%)	
North East & Cumbria	26.5%	(24.0–29.1%)		6.9%	(5.5–8.5%)		20.7%	(18.4–23.1%)		3.9%	(2.9–5.2%)		42.1%	(39.2–45.0%)	
Lancashire & South Cumbria	22.7%	(19.5–26.3%)		14.3%	(11.7–17.5%)		14.0%	(11.4–17.1%)		2.6%	(1.6–4.3%)		46.3%	(42.3–50.4%)	
Greater Manchester	21.8%	(19.4–24.4%)		18.4%	(16.2–20.9%)		13.4%	(11.5–15.6%)		2.3%	(1.6–3.4%)		44.0%	(41.0–47.1%)	
North Central & North East London	15.2%	(13.0–17.8%)		41.6%	(38.3–44.9%)		12.6%	(10.6–15.0%)		2.4%	(1.5–3.6%)		28.2%	(25.3–31.3%)	
North West & South West London	18.6%	(16.1–21.3%)	<0.001	32.6%	(29.6–35.8%)	<0.001	11.6%	(9.6–13.9%)	<0.001	2.4%	(1.5–3.6%)	0.008	34.9%	(31.8–38.1%)	<0.001
*By Cancer Alliance, adjusted for age, sex, deprivation and ethnicity*															
West Yorkshire	20.4%	(17.8–23.3%)		10.3%	(8.5–12.5%)		14.2%	(12.1–16.7%)		1.4%	(0.9–2.2%)		43.7%	(40.2–47.3%)	
Humber, Coast & Vale	17.1%	(13.7–21.1%)		6.4%	(4.3–9.3%)		15.9%	(12.7–19.8%)		1.0%	(0.5–2.1%)		51.0%	(45.7–56.2%)	
Cheshire & Merseyside	23.6%	(21.1–26.4%)		10.6%	(8.8–12.6%)		14.1%	(12.1–16.4%)		2.2%	(1.6–3.1%)		38.5%	(35.3–41.8%)	
South Yorkshire, Bassetlaw, North Derbyshire & Hardwick	14.8%	(12.4–17.6%)		9.1%	(7.2–11.4%)		14.5%	(12.1–17.3%)		1.8%	(1.2–2.7%)		50.7%	(46.7–54.7%)	
West Midlands	19.3%	(17.4–21.3%)		8.7%	(7.5–10.1%)		15.5%	(13.8–17.3%)		1.8%	(1.3–2.4%)		45.1%	(42.6–47.7%)	
East Midlands	15.8%	(13.7–18.1%)		9.9%	(8.2–11.8%)		16.4%	(14.2–18.8%)		2.7%	(2.0–3.7%)		45.2%	(42.0–48.6%)	
East of England	16.0%	(14.3–17.8%)		13.6%	(12.0–15.5%)		16.8%	(15.1–18.7%)		1.8%	(1.3–2.4%)		41.8%	(39.2–44.3%)	
South East London	16.6%	(13.2–20.6%)		18.4%	(15.0–22.4%)		13.1%	(10.0–16.9%)		1.7%	(0.9–3.1%)		37.7%	(32.8–42.8%)	
Kent & Medway	22.3%	(18.6–26.6%)		11.7%	(8.9–15.3%)		15.5%	(12.4–19.3%)		2.1%	(1.2–3.4%)		38.0%	(33.3–43.1%)	
Surrey & Sussex	19.8%	(17.1–22.8%)		15.4%	(12.7–18.4%)		14.7%	(12.4–17.4%)		1.1%	(0.6–1.8%)		39.7%	(36.1–43.4%)	
Thames Valley	20.2%	(17.0–24.0%)		14.4%	(11.5–17.9%)		13.2%	(10.5–16.4%)		0.8%	(0.4–1.6%)		42.3%	(37.8–46.9%)	
Peninsula	20.0%	(16.9–23.6%)		12.4%	(9.7–15.6%)		18.1%	(15.1–21.6%)		1.6%	(1.0–2.7%)		37.1%	(32.8–41.5%)	
Somerset, Wiltshire, Avon & Gloucestershire	18.0%	(15.4–20.8%)		12.8%	(10.4–15.5%)		17.1%	(14.5–19.9%)		1.2%	(0.8–2.0%)		41.7%	(38.0–45.4%)	
Wessex	20.0%	(17.3–22.9%)		11.1%	(8.9–13.6%)		18.2%	(15.7–21.1%)		1.5%	(0.9–2.3%)		39.0%	(35.4–42.6%)	
North East & Cumbria	23.7%	(21.3–26.3%)		5.8%	(4.6–7.3%)		19.1%	(16.9–21.4%)		2.1%	(1.5–2.9%)		38.3%	(35.4–41.4%)	
Lancashire & South Cumbria	19.6%	(16.6–23.0%)		9.6%	(7.6–12.2%)		13.2%	(10.7–16.2%)		1.4%	(0.8–2.4%)		45.9%	(41.6–50.3%)	
Greater Manchester	19.3%	(17.1–21.8%)		11.5%	(9.8–13.5%)		13.0%	(11.1–15.2%)		1.3%	(0.9–2.0%)		44.8%	(41.6–48.1%)	
North Central & North East London	15.5%	(13.1–18.2%)		22.0%	(19.2–25.0%)		14.3%	(11.9–17.0%)		1.9%	(1.2–3.0%)		31.9%	(28.5–35.5%)	
North West & South West London	19.6%	(17.0–22.6%)	<0.001	19.4%	(16.9–22.1%)	<0.001	12.4%	(10.3–14.8%)	0.001	1.5%	(1.0–2.4%)	0.023	36.4%	(33.0–39.9%)	<0.001
Covariate odds ratios (95%CI)															
Per year increase in age	0.97	(0.96–0.97)	<0.001	0.92	(0.91–0.92)	<0.001	1.02	(1.02–1.03)	<0.001	1.01	(1.00–1.02)	0.005	1.06	(1.06–1.06)	<0.001
Female	0.43	(0.38–0.48)	<0.001	0.77	(0.68–0.87)	<0.001	1.11	(1.01–1.23)	0.039	9.73	(7.95–11.92)	<0.001	1.11	(1.02–1.20)	0.013
Per quintile increase in deprivation	1.00	(0.97–1.03)	0.829	1.20	(1.15–1.24)	<0.001	0.97	(0.94–1.00)	0.091	0.88	(0.83–0.95)	<0.001	0.95	(0.93–0.98)	<0.001
Ethnicity															
White	ref			ref			ref			ref			ref		
Black	0.14	(0.09–0.21)	<0.001	3.69	(2.87–4.74)	<0.001	0.28	(0.17–0.47)	<0.001	0.08	(0.01–0.57)	0.012	1.17	(0.92–1.49)	0.213
South Asian	0.28	(0.21–0.37)	<0.001	3.76	(3.12–4.52)	<0.001	1.11	(0.90–1.38)	0.338	0.43	(0.22–0.82)	0.011	0.75	(0.63–0.90)	0.002
Other Asian	0.19	(0.12–0.29)	<0.001	3.75	(2.92–4.81)	<0.001	0.85	(0.61–1.18)	0.324	0.64	(0.29–1.39)	0.257	0.85	(0.66–1.09)	0.193
Other Ethnicity	0.40	(0.28–0.57)	<0.001	3.53	(2.69–4.62)	<0.001	0.77	(0.53–1.12)	0.179	0.53	(0.21–1.31)	0.169	0.68	(0.51–0.90)	0.007
Not Known	0.63	(0.51–0.77)	<0.001	0.85	(0.67–1.09)	0.196	0.47	(0.36–0.61)	<0.001	0.40	(0.22–0.73)	0.003	2.23	(1.89–2.62)	<0.001

### Routes to diagnosis (RTD)

**Fig. 2:**
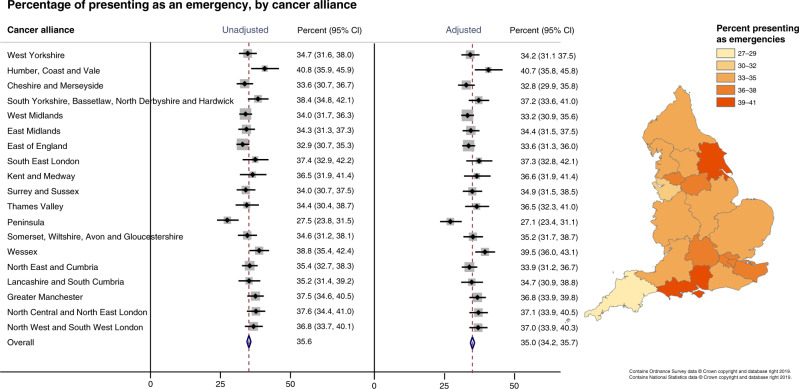
Proportion of patients presenting as an emergency, by Cancer Alliance. Proportions unadjusted and adjusted for age, sex and deprivation quintile shown in the forest plot (left) and adjusted proportions displayed in the choropleth map (right).

In England between 2010 and 2016, the two most common RTDs for HCC were emergency presentation (35.6%) and GP referral (31.1%), followed by other-outpatient (17.2%) and TWW (11.5%) (Supplementary Table 2). There were geographical variations in the proportion of patients presenting through different RTDs, which was not significantly attenuated following adjustment for differences in age, sex and deprivation between Cancer Alliances (Fig. 2 and Supplementary Table 2). After adjustment, emergency presentations, which have the poorest prognosis [17], accounted for 27.1% of diagnoses in Peninsula and 40.7% in Humber, Coast and Vale (*p* for difference between Cancer Alliances 0.002). The odds of an emergency presentation increased 1% per year of age, 10% per quintile increase in deprivation and 19% in women compared to men. The opposite was seen for outpatient presentations, which have a more favourable prognosis; adjusted proportions were lowest in Humber, Coast and Vale (9.0%) and highest in Peninsula (24.9%) (*p* for difference <0.001) and odds were inversely associated with age (−2% per year) and deprivation (−7% per quintile). GP presentations, which also have a better prognosis, ranged from 25.8% in Wessex to 34.8% in both West Yorkshire and East of England (*p* for difference <0.001). Sex, but not deprivation or age, was associated with odds of a GP presentation, with women less likely to present via this route. There was also geographic variation in the proportion presenting through TWW and unknown routes, but not for the inpatient electives. There were insufficient numbers of death certificate only registrations to permit analysis.

### Curative treatment

**Fig. 3:**
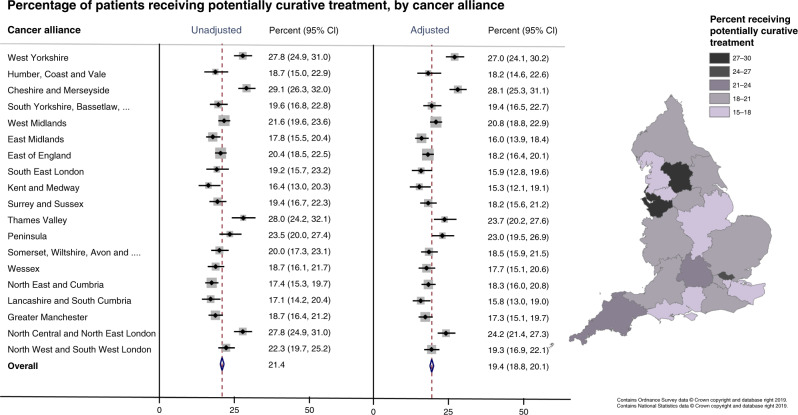
Proportion of patients receiving potentially curative treatments, by Cancer Alliance. Proportions unadjusted and adjusted for age, sex and deprivation quintile shown in the forest plot (left) and adjusted proportions displayed in the choropleth map (right).

Overall, 21.4% of patients received potentially curative treatment between 2010 and 2016. There was wide variation between Cancer Alliances even after adjustment for age, sex and deprivation (p for difference < 0.001) (Fig. 3). Less than 16% of patients in Kent and Medway, Lancashire and South Cumbria, and South East London received potentially curative treatment compared to 27% or more in Cheshire and Merseyside and West Yorkshire. There was a strong negative association between increasing age and deprivation quintile and odds of curative treatment (−5% per year age increase and −11% per deprivation quintile). Women were 11% more likely to receive potentially curative treatment, though the confidence intervals were wide (Supplementary Table 3).

### Survival

**Fig. 4:**
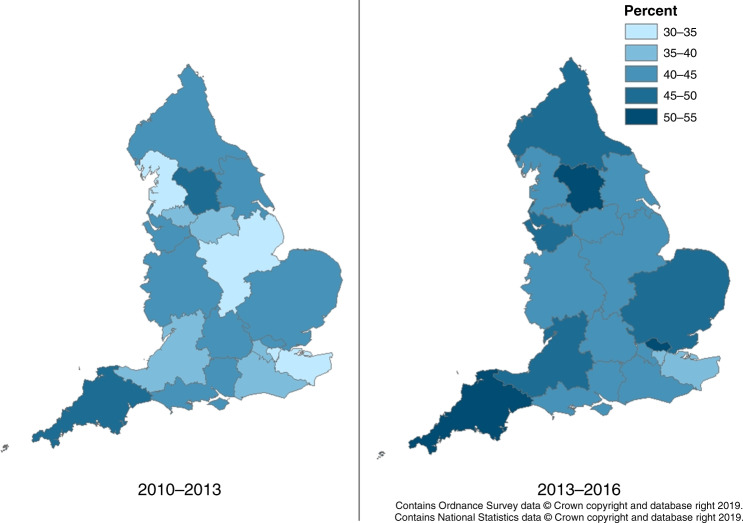
One-year net survival by Cancer Alliance and cohort.

Both 1- and 2-year net survival improved by 5% overall in England between 2010–2013 and 2013–2016 (1-year survival increased from 40.2 to 45.2% and 2-year from 27.8 to 32.8%) (Fig. 4 and Supplementary Tables 4 and 5). There was geographical variation across the nation in the improvement in survival; the largest increase in survival was in North Central and North East London (approximately 10% increase in both 1- and 2-year survival) and there was no clear increase in survival in Wessex and Thames Valley. In the most recent cohort (2013–2016) survival was lowest in Kent and Medway (both 1- and 2-year, 38.2% and 26.2%, respectively) and highest in West Yorkshire (1-year 53.0%, 2-year 39.8%) and Peninsula (1-year 51.1%, 2-year 39.8%).

### Associations between metrics across Cancer Alliances

At the Cancer Alliance level, there was a strong correlation between those Cancer Alliances with a high proportion of the population in the most deprived quintile, such as Greater Manchester, Cheshire and Merseyside, and North Central and North East London and those having high HCC incidence rates (Supplementary Table 6). No strong correlation between Cancer Alliance-level deprivation and survival were seen. Cancer Alliances with a low proportion of emergency presentations, such as Peninsula, and Cheshire and Merseyside, had longer survival. As would be expected, those Cancer Alliances with a high proportion receiving potentially curative treatment also had longer survival, West Yorkshire, Cheshire and Merseyside and North Central and North East London. There was a non-significant negative correlation between Cancer Alliances with higher proportion presenting as emergencies and proportion receiving potentially curative treatment.

## Discussion

### Key findings

The authors believe this to be the first study of regional variations in HCC across England using population-wide high-quality cancer registration data and found HCC incidence rates were nearly two times higher in the highest incident Cancer Alliances compared with the lowest. The high incident areas, predominately in the North of the country and London, generally had higher levels of deprivation. The proportion of cases associated with different PLDs varied widely across the country, particularly for viral hepatitis. Survival increased in most areas across the country during the relatively short study period, varied widely across the country, and was highest in Cancer Alliances with a low proportion of emergency presentations and a high proportion treated with potentially curative treatment. One in five patients received potentially curative treatment, and this ranged from 15 to 28% across all Cancer Alliances and was negatively associated with age and deprivation, more frequent in women.

### Strengths and limitations

The analysis was population-based and includes a large number of HCC patients nationally. It used high quality data from the national cancer registry with near complete population coverage meaning that extrapolation was not needed and consequently, selection biases were minimised. Highly trained cancer registration officers in NCRAS standardise cancer registrations across the country using multiple data sources, allowing accurate between region comparisons [11]. Two nationwide treatment data sources were used to identify potentially curative treatments. There was comprehensive follow up of patients via mortality data from the Office for National Statistics. The age-standardised net survival methodology used allowed comparison of a relatively rare cancer across geographies with potentially different age structures.

However, administrative data were used to help identify treatments and may not capture all relevant diagnoses and treatments accurately. These data were supplemented with the cancer registration treatment data, which are derived from multiple sources, therefore the risk of missing treatment data was minimised. 42% of patients had no PLD identified by our algorithm. We compared our results in North East and Cumbria to chronic liver disease records identified from a clinical database of cases referred to the Newcastle-upon-Tyne Hospitals NHS Foundation Trust HPB MDT between 2000 and 2010 [20] as a gold standard. 21% of patients had no chronic liver disease, 4% had cryptogenic liver disease and 5% other PLDs including haemochromatosis in the clinical dataset, a total of 31%, which when compared to our 42%, indicates our algorithm is detecting the majority of known PLD, at least in this region. There was insufficient data detail or completeness available to include cancer stage, synthetic liver function and performance status in these analyses. This would have been particularly beneficial for improving the classification of curative treatment, which is mainly based on the Barcelona Clinic Liver Cancer (BCLC) stage. Whether a patient was undergoing liver surveillance at the time of diagnosis, an important prognostic factor, was also not known. Surveillance has been found to be associated with improved early stage detection and potentially curative treatment rates [21]. Depending on the referral pathway, patients diagnosed via surveillance are most likely to present via outpatient or GP referral. The majority of HCCs are diagnosed using radiological techniques with high specificity (rather than using histology) in line with EASL clinical guidelines, so a small proportion of non-HCC tumours (e.g. neuroendocrine tumours or metastases or mixed HCC-cholangiocarcinoma), may have been included, as would be the case with any population-based study in a country with similar diagnostic practices. No individual-level measure of deprivation was available and the ecological deprivation measure used, based on the income domain of the index of multiple deprivation allocated on the small area level, may not have accurately captured all socioeconomic differences, therefore the effect of socioeconomic deprivation on regional differences may have been under-estimated.

### Comparison with other studies

Within-country regional differences in HCC incidence, access to treatment and outcomes have been reported previously in France and the USA [9, 10]. Although both are developed countries, they have high HCC incidence rates compared to England, a different distribution of risk factors, different healthcare structures, and the USA has a substantially larger population. In France, liver cancer mortality has been decreasing since approximately 2001, whereas in the USA and UK rates increased substantially [22]. Despite this, and different methodologies used by the studies, the magnitude of the regional difference is strikingly similar. The incidence of HCC was found to vary approximately two-fold across regions of France [10] and census divisions in the USA [9], as it did across Cancer Alliances in England. The proportion of patients receiving potentially curative treatment, albeit within different time periods, was also comparable; 21.4% in England (2010–2016), 22.8% in France (2009–2012) [10] and 23% in the US (2000–2010) [23]. One-year net survival ranged from 38 to 53% across Cancer Alliances in England and regional variations were also found in France, where median survival ranged from 5.7 to 12.1 months across regions [10]. Within-country variation in aetiology (ALD and HCV only) has also been examined in France using electronic health records and found to vary 2- to 3-fold across regions [24]. Overall in France, 44% of HCC cases were related to ALD and 11% to viral hepatitis, compared to 21 and 17% in England, respectively [10].

### Interpretation

The significant geographical variation in incidence worldwide largely reflects the distribution of different aetiological factors which change over time and with socio-demographic shifts, the development and availability of therapies to treat underlying liver disease such as hepatitis C and public health initiatives such as hepatitis B vaccination programmes [2]. Variation within a country, where public health initiatives and therapy availability are more uniform, may be more to do with differences in the prevalence of liver disease risk factors. Here we found large differences in the proportion of HCCs associated with different PLDs across the nation, particularly for viral hepatitis which varied 6-fold. Aetiology affects age at diagnosis and may also influence engagement with specialist liver services including liver surveillance, cirrhosis severity at HCC diagnosis, likelihood of comorbidities and eligibility for transplant and other curative treatments [3, 25]. The association between deprivation and HCC incidence is well established [26–28] and has been found by some studies to be accounted for by risk factors for liver disease, which are closely associated with deprivation [28, 29]. In England, the Atlas of Variation found areas with high deprivation such as central London and North West England have higher rates of hepatitis C, hepatitis B and alcohol-related and cirrhosis-related hospital admissions [5]. We also found a higher proportion of HCCs were related to viral hepatitis in London, that increasing deprivation, younger age at diagnosis and non-white ethnicity were strongly associated with viral hepatitis-related HCC. Adjustment for demographic variables accounted for some of the regional variation seen. The population in London is younger and more ethnically diverse with a larger proportion of migrants than many other parts of England [30, 31]. Whilst information on migration status was not available, the majority of chronic HBV infection in England occurs in migrants from HBV endemic countries [32, 33]. Ethnicity is strongly associated with deprivation in the UK [34]. In addition, intravenous drug use is the leading risk factor for HCV in the UK [35], increases risk of HBV [32], and is associated with deprivation [36]. We did not see a strong association between ALD-related HCC and deprivation, but found the highest proportion of ALD-related HCC be to in the North, and that this was associated with white ethnicity, male sex and younger age at diagnosis. There was less geographical variation in NAFLD-associated HCC, and it was not strongly associated with deprivation.

Emergency presentation, which has the poorest prognosis [17], was the most common route to diagnosis for HCC cases at 30%; higher than for most cancers [17]. Patients presenting with emergency symptoms of decompensated liver disease, i.e. ascites, bleeding, infection and encephalopathy, may have their HCC diagnosed incidentally during diagnosis and treatment for decompensation. Socioeconomic deprivation, increasing age, and female gender were associated with higher chance of emergency presentation, but these factors alone did not explain the large regional variation. Individual-level factors associated with deprivation not captured here, such as liver disease risk factors including smoking and alcohol use, liver disease severity, education, and health seeking behaviours may contribute, as could proximity to and accessibility of specialist liver services [37]. Increasing socioeconomic deprivation and age were associated with a reduced likelihood of receiving potentially curative treatment, whereas, female gender was associated with an increased likelihood. Access to and suitability for treatment may be inhibited by extent of tumour burden, co-morbidities, aetiology and degree of underlying liver disease on the individual-level, and on the provision of specialist liver services at the regional level. A clear association between regions with high proportions receiving potentially curative treatment and longer survival was seen in the present study and by Goutte et al. [10]. A thorough exploration of potential factors associated with regional variation in treatment rates and survival is beyond the scope of this paper but will be explored by future HCC-UK BASL/NCRAS partnership work.

## Conclusion

Despite nationwide universal healthcare that is free at the point of delivery, regional variations in HCC incidence, care and survival across England exist and socioeconomic inequality may be a significant factor in this. This work provides contemporary data to assist health policy makers to target interventions to prevent HCC and improve outcomes in those who do develop it. Further work to explore the causes, and identify potential targeted interventions are needed to ensure equal, high quality care across the country.

## Supplementary information

HCC-UK/NCRAS Steering Group

Supplementary Information

Reproduction checklist

STROBE Checklist

## Data Availability

Access to cancer registration data through Public Health England can be granted provided there is a justified purpose for the data release, and that there is an appropriate legal basis with safeguards in place to protect the data. Dependent on the request, ethical approval may be required. The access process is managed by the Office for Data Release.
