# Supplementary material for: Regional variations in hepatocellular carcinoma incidence, routes to diagnosis, treatment and survival in England
Source: Br J Cancer. 2021 Nov 26;126(5):804–14. doi: 10.1038/s41416-021-01509-4 (PMC8888669; doi:10.1038/s41416-021-01509-4)
Supplement: Supplementary file 1 — HCC-UK/NCRAS Steering Group [file 41416_2021_1509_MOESM1_ESM.docx]

**HCC-UK BASL/NCRAS Partnership steering group members**

Anya Burton^1,2^, Aileen Marshall^5^, Graeme Alexander^6^, Ian A Rowe^4^, Robert J Driver^4^, Vinay K Balachandrakumar^3^, Tim J S Cross^3^,Katherine Cullen^8^, Tom Bird^9, 10,11^ , Dyfed W Huws ^12^, Anna Gavin^13^, David Wallace^14^, Daniela Tataru^2^, Lizz Paley^2^

1 HCC-UK/British Association for the Study of the Liver

2 National Cancer Registration and Analysis Service (NCRAS), Public Health England, London , UK,

3 Department of Molecular and Clinical Cancer Medicine**,** Institute of Translational medicine, University of Liverpool, Liverpool, UK

4 Leeds Institute for Medical Research at St. James’s, University of Leeds, Leeds, UK

5 Sheila Sherlock Liver Centre, The Royal Free Hospital, London, UK

6 Institute for Liver and Digestive Health, Royal Free Hospital Pond St, Hampstead, London, UK

7 Liverpool Experimental Cancer Medicine Centre, University of Liverpool, Liverpool, UK

8 Swansea Centre for Health Economics, Swansea University, Singleton Campus, Swansea, Wales, UK

9 Cancer Research UK Beatson Institute, Garscube Estate, Switchback Road, Glasgow, Scotland, UK

10 Institute of Cancer Sciences, University of Glasgow, Garscube Estate, Switchback Road, Glasgow, Scotland, UK

11 MRC Centre for Inflammation Research, University of Edinburgh, Edinburgh, Scotland, UK

12 Welsh Cancer Intelligence and Surveillance Unit, Public Health Wales, 2 Capital Quarter, Tyndall Street, Cardiff, Wales, UK

13 Northern Ireland Cancer Registry, Centre for Public Health, Mulhouse Building, RVH, Grosvenor Road, Belfast, Northern Ireland, UK

14 Department of Health Services Research and Policy, London School of Hygiene and Tropical Medicine, 15-17 Tavistock Place, London, England, UK
