# Supplementary material for: Regional variations in hepatocellular carcinoma incidence, routes to diagnosis, treatment and survival in England
Source: Br J Cancer. 2021 Nov 26;126(5):804–14. doi: 10.1038/s41416-021-01509-4 (PMC8888669; doi:10.1038/s41416-021-01509-4)
Supplement: Supplementary file 2 — Supplementary Information [file 41416_2021_1509_MOESM2_ESM.docx]

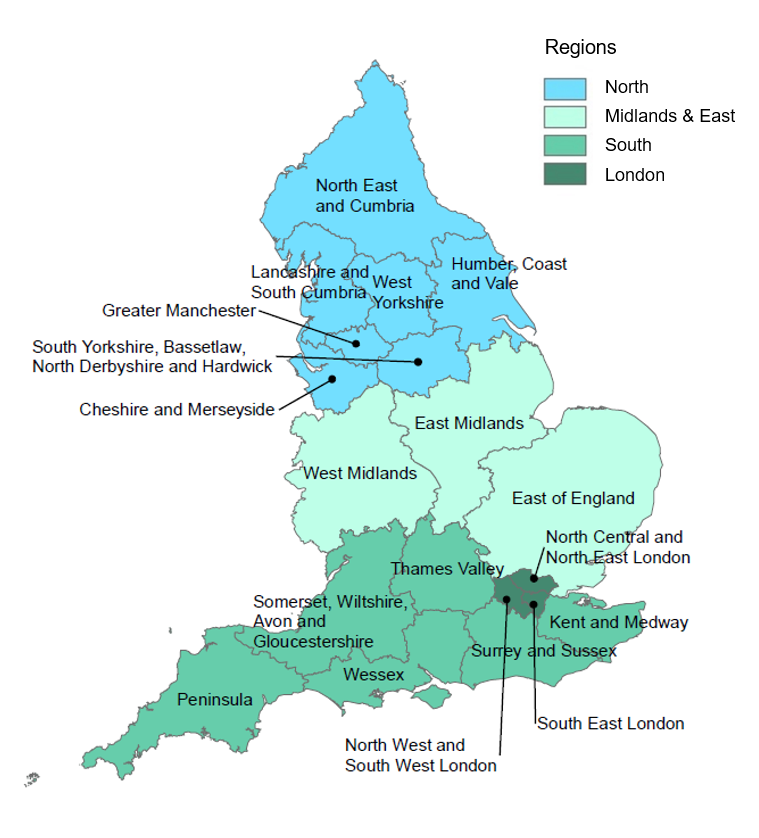


**Supplementary Figure 1 Cancer Alliances in England 2017, by region**


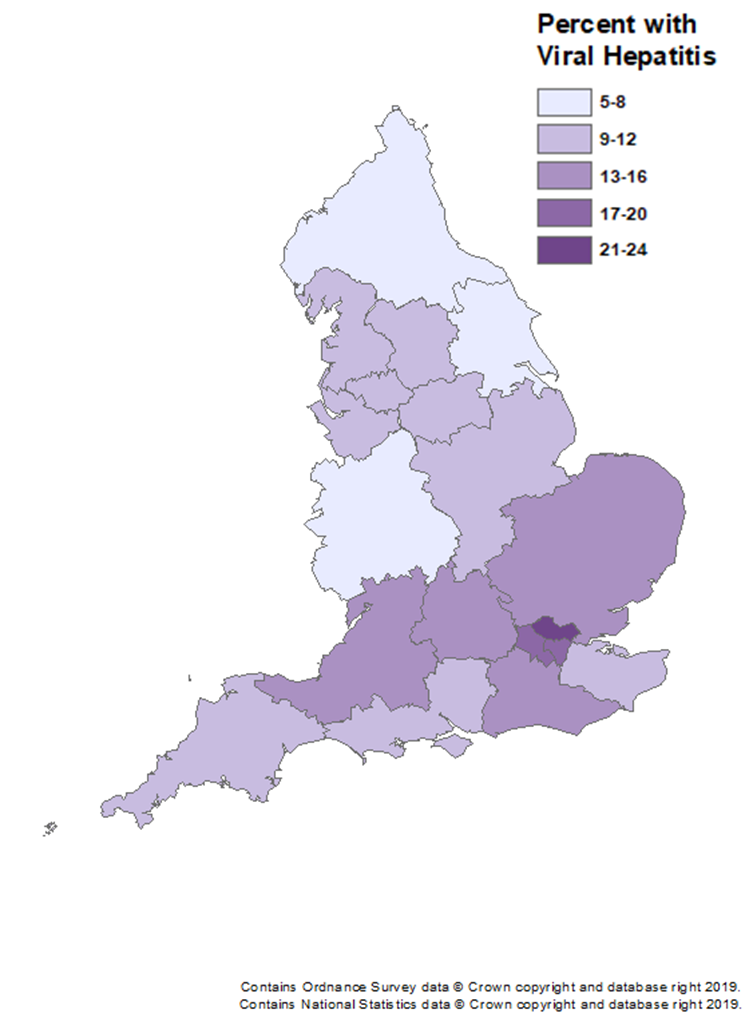


**Supplementary Figure 2: Proportion of patients with viral hepatitis, by Cancer Alliance, adjusted for age, sex, deprivation quintile and ethnicity**

**Supplementary Table 1: European age-standardised HCC incidence rate by year, sex and Cancer Alliance**

|  | **Year** | | | | | | | | | | | | | |
| --- | --- | --- | --- | --- | --- | --- | --- | --- | --- | --- | --- | --- | --- | --- |
|  | **2010** | | **2011** | | **2012** | | **2013** | | **2014** | | **2015** | | **2016** | |
|  | ASR | 95% CI | ASR | 95% CI | ASR | 95% CI | ASR | 95% CI | ASR | 95% CI | ASR | 95% CI | ASR | 95% CI |
| **Men** |  |  |  |  |  |  |  |  |  |  |  |  |  |  |
| England Overall | 6.2 | (5.9-6.5) | 6.4 | (6.1-6.8) | 7.2 | (6.9-7.6) | 8.1 | (7.7-8.5) | 8.6 | (8.2-9) | 8.5 | (8.1-8.9) | 8.8 | (8.4-9.2) |
| West Yorkshire | 8.4 | (6.6-10.6) | 8.0 | (6.3-10.1) | 8.0 | (6.2-10) | 9.0 | (7.3-11.1) | 11.6 | (9.5-14) | 11.8 | (9.7-14.2) | 11.3 | (9.3-13.7) |
| Humber, Coast and Vale | 6.1 | (4.2-8.5) | 5.5 | (3.8-7.8) | 4.5 | (3-6.5) | 6.8 | (4.8-9.2) | 7.6 | (5.6-10) | 7.4 | (5.5-9.8) | 9.1 | (6.9-11.7) |
| Cheshire and Merseyside | 8.1 | (6.5-10.1) | 8.7 | (7-10.7) | 8.1 | (6.4-10) | 10.3 | (8.4-12.5) | 11.0 | (9.1-13.1) | 10.4 | (8.6-12.5) | 10.3 | (8.4-12.3) |
| South Yorkshire, Bassetlaw, North Derbyshire and Hardwick | 6.8 | (5.1-8.9) | 9.3 | (7.3-11.8) | 7.1 | (5.3-9.2) | 8.9 | (6.9-11.2) | 9.7 | (7.6-12) | 9.8 | (7.8-12.1) | 11.5 | (9.3-14) |
| West Midlands | 5.4 | (4.5-6.4) | 6.5 | (5.5-7.6) | 6.7 | (5.7-7.8) | 8.4 | (7.2-9.6) | 7.9 | (6.8-9.1) | 7.8 | (6.7-9) | 8.4 | (7.3-9.7) |
| East Midlands | 3.7 | (2.8-4.7) | 4.9 | (3.9-6.1) | 5.7 | (4.6-7) | 6.7 | (5.5-8) | 6.8 | (5.7-8.2) | 7.3 | (6.1-8.6) | 6.8 | (5.6-8.1) |
| East of England | 6.0 | (5.1-7) | 5.8 | (4.9-6.8) | 6.7 | (5.7-7.7) | 6.1 | (5.2-7.1) | 6.4 | (5.5-7.5) | 7.2 | (6.2-8.3) | 7.3 | (6.3-8.3) |
| South East London | 10.1 | (7.5-13.3) | 7.9 | (5.7-10.8) | 9.8 | (7.2-13) | 6.8 | (4.7-9.5) | 8.1 | (5.7-11.1) | 9.9 | (7.4-13) | 7.9 | (5.6-10.7) |
| Kent and Medway | 4.9 | (3.4-6.8) | 3.9 | (2.6-5.6) | 5.3 | (3.7-7.2) | 4.1 | (2.7-5.8) | 7.0 | (5.3-9) | 6.5 | (4.8-8.5) | 7.1 | (5.4-9.1) |
| Surrey and Sussex | 5.1 | (3.9-6.5) | 4.7 | (3.6-6) | 6.0 | (4.7-7.5) | 6.5 | (5.2-8.1) | 7.0 | (5.7-8.6) | 7.0 | (5.7-8.6) | 8.4 | (7-10.1) |
| Thames Valley | 4.3 | (3-5.9) | 3.7 | (2.5-5.3) | 6.2 | (4.6-8.1) | 5.8 | (4.3-7.6) | 7.3 | (5.6-9.3) | 7.4 | (5.7-9.3) | 7.6 | (5.9-9.5) |
| Peninsula | 4.1 | (2.8-5.7) | 6.0 | (4.5-8) | 5.5 | (4-7.3) | 7.5 | (5.8-9.6) | 6.4 | (4.8-8.3) | 8.4 | (6.6-10.5) | 8.4 | (6.6-10.5) |
| Somerset, Wiltshire, Avon and Gloucestershire | 3.6 | (2.6-4.8) | 5.7 | (4.4-7.2) | 7.1 | (5.7-8.8) | 6.2 | (4.9-7.8) | 7.8 | (6.3-9.5) | 7.7 | (6.2-9.4) | 7.7 | (6.2-9.3) |
| Wessex | 3.9 | (2.8-5.2) | 4.6 | (3.4-6.1) | 9.0 | (7.3-10.9) | 7.6 | (6.1-9.4) | 8.6 | (7-10.4) | 7.0 | (5.6-8.7) | 9.1 | (7.5-10.9) |
| North East and Cumbria | 9.1 | (7.5-11) | 7.4 | (6-9.1) | 8.3 | (6.7-10) | 11.7 | (9.9-13.7) | 10.7 | (9-12.6) | 8.8 | (7.3-10.5) | 8.7 | (7.2-10.4) |
| Lancashire and South Cumbria | 4.4 | (2.9-6.3) | 7.3 | (5.4-9.7) | 8.5 | (6.5-10.8) | 9.1 | (7-11.5) | 10.1 | (7.9-12.6) | 8.9 | (6.9-11.2) | 10.2 | (8.1-12.7) |
| Greater Manchester | 9.8 | (7.8-12.1) | 7.0 | (5.4-8.9) | 9.8 | (8-12) | 13.5 | (11.3-16) | 11.7 | (9.7-14) | 13.8 | (11.6-16.2) | 12.1 | (10.1-14.4) |
| North Central and North East London | 9.7 | (7.7-12.1) | 9.5 | (7.6-11.8) | 8.4 | (6.6-10.5) | 9.8 | (7.8-12.1) | 12.6 | (10.4-15.1) | 9.7 | (7.8-12) | 10.5 | (8.5-12.7) |
| North West and South West London | 7.6 | (6-9.5) | 7.8 | (6.2-9.7) | 7.6 | (6-9.5) | 10.1 | (8.2-12.2) | 9.3 | (7.5-11.3) | 8.6 | (6.9-10.6) | 9.0 | (7.3-11) |
|  |  |  |  |  |  |  |  |  |  |  |  |  |  |  |
| **Women** |  |  |  |  |  |  |  |  |  |  |  |  |  |  |
| England Overall | 1.5 | (1.3-1.6) | 1.7 | (1.5-1.9) | 1.8 | (1.6-2) | s | (1.9-2.2) | 1.9 | (1.8-2.1) | 2.1 | (1.9-2.3) | 2.2 | (2-2.4) |
| West Yorkshire | 2.4 | (1.6-3.5) | 2.1 | (1.4-3.2) | 2.4 | (1.6-3.5) | 2.5 | (1.7-3.6) | 2.4 | (1.6-3.5) | 2.3 | (1.5-3.3) | 2.6 | (1.7-3.6) |
| Humber, Coast and Vale | 1.4 | (0.7-2.5) | 2.2 | (1.3-3.7) | 1.9 | (1-3.2) | 1.5 | (0.8-2.6) | 1.6 | (0.8-2.7) | 1.3 | (0.6-2.4) | 0.9 | (0.4-1.9) |
| Cheshire and Merseyside | 1.9 | (1.2-2.8) | 2.7 | (1.8-3.8) | 2.2 | (1.4-3.2) | 3.6 | (2.6-4.8) | 2.8 | (2-3.9) | 2.8 | (2-3.9) | 2.6 | (1.8-3.6) |
| South Yorkshire, Bassetlaw, North Derbyshire and Hardwick | 1.8 | (1-2.9) | 3.6 | (2.5-5) | 1.5 | (0.9-2.5) | 2.2 | (1.4-3.4) | 2.0 | (1.2-3.1) | 2.4 | (1.5-3.6) | 3.3 | (2.2-4.6) |
| West Midlands | 1.2 | (0.8-1.7) | 2.1 | (1.6-2.7) | 1.5 | (1.1-2) | 2.4 | (1.8-3) | 1.9 | (1.4-2.4) | 2.3 | (1.8-2.9) | 1.7 | (1.2-2.2) |
| East Midlands | 1.0 | (0.6-1.6) | 1.2 | (0.8-1.8) | 1.5 | (1-2.2) | 1.6 | (1.1-2.2) | 1.6 | (1.1-2.2) | 2.1 | (1.5-2.8) | 1.7 | (1.2-2.4) |
| East of England | 1.3 | (0.9-1.7) | 1.7 | (1.3-2.2) | 1.8 | (1.3-2.3) | 1.7 | (1.3-2.2) | 1.4 | (1.1-1.9) | 1.8 | (1.4-2.3) | 1.7 | (1.3-2.2) |
| South East London | 3.0 | (1.7-4.8) | 1.3 | (0.5-2.6) | 2 | (1-3.6) | 2.0 | (1.1-3.5) | 2.5 | (1.4-4.2) | 1.7 | (0.9-3) | 3.4 | (2.1-5.3) |
| Kent and Medway | 0.3 | (0.1-0.9) | 1.6 | (0.9-2.7) | 1.2 | (0.6-2.1) | 1.2 | (0.6-2.2) | 2.1 | (1.2-3.2) | 2.4 | (1.5-3.6) | 1.6 | (0.9-2.7) |
| Surrey and Sussex | 1.1 | (0.7-1.8) | 1.3 | (0.8-2) | 1.5 | (1-2.2) | 1.3 | (0.8-2) | 0.9 | (0.5-1.5) | 1.0 | (0.6-1.6) | 2.0 | (1.4-2.8) |
| Thames Valley | 0.8 | (0.4-1.7) | 0.7 | (0.3-1.5) | 2.1 | (1.3-3.2) | 1.2 | (0.7-2.1) | 1.7 | (1-2.6) | 1.8 | (1.1-2.8) | 2.1 | (1.3-3.1) |
| Peninsula | 0.7 | (0.2-1.4) | 0.9 | (0.4-1.7) | 2.4 | (1.6-3.6) | 1.2 | (0.6-2.1) | 1.7 | (1-2.7) | 1.8 | (1.1-2.8) | 1.6 | (0.9-2.6) |
| Somerset, Wiltshire, Avon and Gloucestershire | 1.3 | (0.8-2.1) | 1.1 | (0.6-1.8) | 1.6 | (1-2.4) | 2.2 | (1.5-3.2) | 1.4 | (0.9-2.2) | 1.8 | (1.2-2.6) | 2.1 | (1.4-3) |
| Wessex | 1.0 | (0.5-1.7) | 0.9 | (0.5-1.6) | 1.5 | (1-2.3) | 1.6 | (1-2.5) | 2.2 | (1.5-3.1) | 1.8 | (1.2-2.7) | 1.9 | (1.2-2.8) |
| North East and Cumbria | 2.2 | (1.5-3.1) | 1.6 | (1.1-2.4) | 1.9 | (1.3-2.7) | 2.0 | (1.4-2.8) | 2.8 | (2-3.7) | 1.8 | (1.2-2.6) | 2.0 | (1.4-2.8) |
| Lancashire and South Cumbria | 0.2 | (0-0.8) | 1.2 | (0.6-2.2) | 1.6 | (0.8-2.6) | 2.5 | (1.6-3.8) | 2.1 | (1.2-3.2) | 3.0 | (1.9-4.3) | 3.6 | (2.4-5) |
| Greater Manchester | 1.8 | (1.1-2.8) | 2.5 | (1.7-3.6) | 1.9 | (1.2-2.9) | 3.8 | (2.8-5.1) | 2.2 | (1.4-3.2) | 2.9 | (2-4) | 2.9 | (2.1-4.1) |
| North Central and North East London | 2.3 | (1.5-3.5) | 1.6 | (0.9-2.5) | 1.9 | (1.1-2.9) | 1.8 | (1.1-2.9) | 3.2 | (2.2-4.4) | 2.4 | (1.6-3.6) | 2.0 | (1.3-3.1) |
| North West and South West London | 2.6 | (1.8-3.7) | 1.8 | (1.1-2.8) | 2 | (1.3-3) | 2.1 | (1.4-3.1) | 1.6 | (1-2.5) | 2.6 | (1.8-3.7) | 3.9 | (2.9-5.1) |

**Supplementary Table 2: Proportion presenting via each route to diagnosis by Cancer Alliance**

| **Route to Diagnosis** | **Unknown** | | **TWW** | | **Outpatient** | | **Inpatient** | | **GP** | | **Emergency** | |
| --- | --- | --- | --- | --- | --- | --- | --- | --- | --- | --- | --- | --- |
|  | Percent | 95% CI | Percent | 95% CI | Percent | 95% CI | Percent | 95% CI | Percent | 95% CI | Percent | 95% CI |
| England Overall | 3.5% |  | 11.5% |  | 17.2% |  | 1.1% |  | 31.1% |  | 35.6% |  |
| **By Cancer Alliance, Unadjusted** |  |  |  |  |  |  |  |  |  |  |  |  |
| West Yorkshire | 3.1% | (2.1%-4.5%) | 12.3% | (10.3%-14.7%) | 14.2% | (12.0%-16.7%) | 0.9% | (0.5%-1.9%) | 34.7% | (31.6%-38.0%) | 34.7% | (31.6%-38.0%) |
| Humber, Coast and Vale | 2.9% | (1.6%-5.2%) | 14.7% | (11.4%-18.6%) | 9.1% | (6.5%-12.4%) | 1.6% | (0.7%-3.5%) | 30.9% | (26.5%-35.8%) | 40.8% | (35.9%-45.9%) |
| Cheshire and Merseyside | 2.9% | (2.0%-4.2%) | 11.2% | (9.4%-13.4%) | 17.7% | (15.4%-20.2%) | 0.5% | (0.2%-1.2%) | 34.0% | (31.0%-37.0%) | 33.6% | (30.7%-36.7%) |
| South Yorkshire, Bassetlaw, North Derbyshire and Hardwick | 1.6% | (0.9%-3.0%) | 15.0% | (12.5%-17.9%) | 18.3% | (15.5%-21.4%) | 0.4% | (0.1%-1.4%) | 25.6% | (22.5%-29.1%) | 38.4% | (34.8%-42.1%) |
| West Midlands | 3.1% | (2.3%-4.0%) | 10.2% | (8.8%-11.8%) | 17.5% | (15.8%-19.5%) | 0.6% | (0.3%-1.1%) | 34.4% | (32.1%-36.8%) | 34.0% | (31.7%-36.3%) |
| East Midlands | 2.8% | (1.9%-4.1%) | 14.0% | (11.9%-16.3%) | 16.1% | (14.0%-18.6%) | 1.5% | (0.9%-2.4%) | 30.9% | (28.1%-33.9%) | 34.3% | (31.3%-37.3%) |
| East of England | 5.5% | (4.5%-6.7%) | 11.1% | (9.7%-12.7%) | 13.9% | (12.3%-15.7%) | 1.6% | (1.1%-2.3%) | 35.0% | (32.7%-37.4%) | 32.9% | (30.7%-35.3%) |
| South East London | 2.9% | (1.6%-5.0%) | 9.4% | (6.9%-12.5%) | 17.0% | (13.7%-20.9%) | 0.7% | (0.2%-2.2%) | 31.4% | (27.1%-36.0%) | 37.4% | (32.9%-42.2%) |
| Kent and Medway | 2.3% | (1.2%-4.3%) | 13.1% | (10.1%-16.8%) | 18.1% | (14.6%-22.2%) | 1.5% | (0.7%-3.3%) | 27.7% | (23.5%-32.3%) | 36.5% | (31.9%-41.4%) |
| Surrey and Sussex | 5.3% | (3.9%-7.2%) | 9.6% | (7.7%-11.9%) | 15.9% | (13.4%-18.7%) | 0.9% | (0.4%-1.9%) | 34.0% | (30.7%-37.5%) | 34.0% | (30.7%-37.5%) |
| Thames Valley | 3.0% | (1.8%-4.9%) | 9.5% | (7.2%-12.4%) | 19.5% | (16.3%-23.2%) | 1.0% | (0.4%-2.4%) | 32.0% | (28.0%-36.2%) | 34.4% | (30.4%-38.7%) |
| Peninsula | 3.5% | (2.2%-5.5%) | 12.4% | (9.8%-15.5%) | 24.9% | (21.3%-28.8%) | 0.6% | (0.2%-1.8%) | 31.0% | (27.1%-35.1%) | 27.5% | (23.8%-31.5%) |
| Somerset, Wiltshire, Avon and Gloucestershire | 2.2% | (1.3%-3.6%) | 16.0% | (13.6%-18.9%) | 17.4% | (14.8%-20.3%) | 1.1% | (0.5%-2.2%) | 28.3% | (25.1%-31.6%) | 34.6% | (31.2%-38.1%) |
| Wessex | 4.4% | (3.2%-6.1%) | 11.1% | (9.0%-13.6%) | 17.3% | (14.7%-20.1%) | 1.9% | (1.1%-3.1%) | 26.0% | (23.0%-29.2%) | 38.8% | (35.4%-42.4%) |
| North East and Cumbria | 4.0% | (3.0%-5.3%) | 12.1% | (10.3%-14.1%) | 16.9% | (14.8%-19.2%) | 1.4% | (0.9%-2.3%) | 30.1% | (27.5%-32.9%) | 35.4% | (32.7%-38.3%) |
| Lancashire and South Cumbria | 2.8% | (1.7%-4.5%) | 14.8% | (12.1%-18.0%) | 15.3% | (12.6%-18.5%) | 1.8% | (0.9%-3.2%) | 29.8% | (26.1%-33.6%) | 35.2% | (31.4%-39.2%) |
| Greater Manchester | 2.4% | (1.7%-3.6%) | 12.9% | (10.9%-15.0%) | 16.2% | (14.0%-18.5%) | 1.1% | (0.6%-1.9%) | 29.7% | (27.0%-32.6%) | 37.5% | (34.6%-40.5%) |
| North Central and North East London | 4.0% | (2.8%-5.5%) | 7.3% | (5.7%-9.3%) | 20.5% | (17.9%-23.4%) | 1.3% | (0.7%-2.4%) | 28.5% | (25.6%-31.7%) | 37.6% | (34.4%-41.0%) |
| North West and South West London | 4.0% | (2.9%-5.6%) | 6.4% | (5.0%-8.3%) | 23.6% | (20.9%-26.5%) | 1.2% | (0.6%-2.1%) | 26.7% | (23.9%-29.7%) | 36.8% | (33.7%-40.1%) |
| p for difference | <0.001 |  | <0.001 |  | <0.001 |  | 0.09 |  | <0.001 |  | 0.002 |  |
|  |  |  |  |  |  |  |  |  |  |  |  |  |
| **By Cancer Alliance Adjusted for age, sex and deprivation quintile** |  |  |  |  |  |  |  |  |  |  |  |  |
| West Yorkshire | 3.1% | (2.1%-4.5%) | 12.0% | (9.9%-14.3%) | 14.0% | (11.8%-16.5%) | 0.9% | (0.5%-1.8%) | 34.8% | (31.7%-38.1%) | 34.2% | (31.1%-37.5%) |
| Humber, Coast and Vale | 2.9% | (1.6%-5.2%) | 13.8% | (10.7%-17.6%) | 9.0% | (6.5%-12.4%) | 1.6% | (0.7%-3.5%) | 31.0% | (26.5%-35.8%) | 40.7% | (35.8%-45.8%) |
| Cheshire and Merseyside | 2.9% | (2.0%-4.2%) | 11.2% | (9.4%-13.4%) | 17.5% | (15.2%-20.0%) | 0.5% | (0.2%-1.2%) | 34.1% | (31.2%-37.2%) | 32.8% | (29.9%-35.8%) |
| South Yorkshire, Bassetlaw, North Derbyshire and Hardwick | 1.7% | (0.9%-3.0%) | 14.4% | (12.0%-17.3%) | 18.6% | (15.8%-21.8%) | 0.5% | (0.1%-1.4%) | 25.9% | (22.7%-29.4%) | 37.2% | (33.6%-41.0%) |
| West Midlands | 3.1% | (2.3%-4.1%) | 9.9% | (8.5%-11.5%) | 17.5% | (15.7%-19.5%) | 0.6% | (0.3%-1.1%) | 34.6% | (32.3%-36.9%) | 33.2% | (30.9%-35.6%) |
| East Midlands | 2.7% | (1.9%-4.0%) | 13.7% | (11.6%-16.0%) | 15.6% | (13.4%-18.0%) | 1.4% | (0.8%-2.3%) | 30.8% | (28.0%-33.8%) | 34.4% | (31.5%-37.5%) |
| East of England | 5.3% | (4.3%-6.4%) | 10.7% | (9.3%-12.3%) | 13.2% | (11.7%-14.9%) | 1.5% | (1.0%-2.2%) | 34.8% | (32.5%-37.1%) | 33.6% | (31.3%-36.0%) |
| South East London | 2.8% | (1.6%-4.9%) | 9.8% | (7.2%-13.1%) | 15.9% | (12.8%-19.7%) | 0.7% | (0.2%-2.0%) | 31.2% | (27.0%-35.9%) | 37.3% | (32.8%-42.1%) |
| Kent and Medway | 2.2% | (1.2%-4.2%) | 12.7% | (9.8%-16.3%) | 17.8% | (14.4%-21.9%) | 1.5% | (0.7%-3.2%) | 27.7% | (23.5%-32.3%) | 36.6% | (31.9%-41.4%) |
| Surrey and Sussex | 5.2% | (3.8%-7.0%) | 8.8% | (7.0%-11.0%) | 15.5% | (13.1%-18.3%) | 0.9% | (0.4%-1.9%) | 33.8% | (30.5%-37.3%) | 34.9% | (31.5%-38.5%) |
| Thames Valley | 2.8% | (1.7%-4.6%) | 9.1% | (6.9%-12.0%) | 17.8% | (14.7%-21.3%) | 0.9% | (0.4%-2.1%) | 31.3% | (27.4%-35.6%) | 36.5% | (32.3%-41.0%) |
| Peninsula | 3.5% | (2.2%-5.5%) | 11.8% | (9.3%-14.8%) | 24.9% | (21.4%-28.9%) | 0.6% | (0.2%-1.8%) | 31.0% | (27.2%-35.2%) | 27.1% | (23.4%-31.1%) |
| Somerset, Wiltshire, Avon and Gloucestershire | 2.1% | (1.3%-3.4%) | 15.3% | (12.9%-18.0%) | 16.9% | (14.4%-19.8%) | 1.0% | (0.5%-2.1%) | 28.1% | (24.9%-31.5%) | 35.2% | (31.7%-38.7%) |
| Wessex | 4.3% | (3.1%-6.0%) | 10.4% | (8.4%-12.7%) | 16.9% | (14.4%-19.8%) | 1.8% | (1.1%-3.0%) | 25.8% | (22.8%-29.1%) | 39.5% | (36.0%-43.1%) |
| North East and Cumbria | 4.1% | (3.1%-5.5%) | 11.4% | (9.7%-13.4%) | 17.6% | (15.4%-20.0%) | 1.5% | (0.9%-2.4%) | 30.5% | (27.9%-33.3%) | 33.9% | (31.2%-36.7%) |
| Lancashire and South Cumbria | 2.8% | (1.7%-4.5%) | 14.5% | (11.8%-17.6%) | 15.1% | (12.4%-18.3%) | 1.7% | (0.9%-3.2%) | 29.8% | (26.2%-33.7%) | 34.7% | (30.9%-38.8%) |
| Greater Manchester | 2.4% | (1.7%-3.6%) | 12.8% | (10.9%-15.0%) | 15.9% | (13.8%-18.3%) | 1.0% | (0.6%-1.9%) | 29.8% | (27.0%-32.6%) | 36.8% | (33.9%-39.8%) |
| North Central and North East London | 4.0% | (2.8%-5.5%) | 7.8% | (6.1%-9.9%) | 19.4% | (16.8%-22.2%) | 1.2% | (0.7%-2.2%) | 28.4% | (25.4%-31.6%) | 37.1% | (33.9%-40.5%) |
| North West and South West London | 3.9% | (2.8%-5.4%) | 6.6% | (5.1%-8.5%) | 22.5% | (19.8%-25.3%) | 1.1% | (0.6%-2.0%) | 26.5% | (23.7%-29.5%) | 37.0% | (33.9%-40.3%) |
| p for difference | <0.001 |  | <0.001 |  | <0.001 |  | 0.11 |  | <0.001 |  | 0.002 |  |
| Covariate odds ratios |  |  |  |  |  |  |  |  |  |  |  |  |
| Per year increase in age | 0.99 | (0.98-1) | 1.02 | (1.02-1.03) | 0.98 | (0.97-0.98) | 0.98 | (0.97-0.99) | 0.99 | (0.99-1) | 1.01 | (1.01-1.02) |
| Female | 1.1 | (0.89-1.35) | 0.79 | (0.7-0.9) | 0.99 | (0.9-1.1) | 0.76 | (0.51-1.14) | 0.91 | (0.84-0.99) | 1.19 | (1.1-1.29) |
| Per quintile increase in deprivation quintile* | 0.93 | (0.87-0.99) | 0.97 | (0.94-1.01) | 0.93 | (0.9-0.96) | 0.94 | (0.84-1.05) | 0.97 | (0.95-1) | 1.1 | (1.08-1.13) |

*Based on the income domain of the Index of Multiple Deprivation

**Supplementary Table 3: Proportion receiving potentially curative treatment by Cancer Alliance**

|  | **Percent receiving potentially curative treatment** | | | |
| --- | --- | --- | --- | --- |
| **Curative Treatment** | **Unadjusted** | | **Adjusted for age, sex and deprivation quintile** | |
|  | Percent | 95% CI | Percent | 95% CI |
| England Overall | 21.4% |  | 19.4% | (18.8%-20.1%) |
| West Yorkshire | 27.8% | (24.9%-31.0%) | 27.0% | (24.1%-30.2%) |
| Humber, Coast and Vale | 18.7% | (15.0%-22.9%) | 18.2% | (14.6%-22.6%) |
| Cheshire and Merseyside | 29.1% | (26.3%-32.0%) | 28.1% | (25.3%-31.1%) |
| South Yorkshire, Bassetlaw, North Derbyshire and Hardwick | 19.6% | (16.8%-22.8%) | 19.4% | (16.5%-22.7%) |
| West Midlands | 21.6% | (19.6%-23.6%) | 20.8% | (18.8%-22.9%) |
| East Midlands | 17.8% | (15.5%-20.4%) | 16.0% | (13.9%-18.4%) |
| East of England | 20.4% | (18.5%-22.5%) | 18.2% | (16.4%-20.1%) |
| South East London | 19.2% | (15.7%-23.2%) | 15.9% | (12.8%-19.6%) |
| Kent and Medway | 16.4% | (13.0%-20.3%) | 15.3% | (12.1%-19.1%) |
| Surrey and Sussex | 19.4% | (16.7%-22.3%) | 18.2% | (15.6%-21.2%) |
| Thames Valley | 28.0% | (24.2%-32.1%) | 23.7% | (20.2%-27.6%) |
| Peninsula | 23.5% | (20.0%-27.4%) | 23.0% | (19.5%-26.9%) |
| Somerset, Wiltshire, Avon and Gloucestershire | 20.0% | (17.3%-23.1%) | 18.5% | (15.9%-21.5%) |
| Wessex | 18.7% | (16.1%-21.7%) | 17.7% | (15.1%-20.6%) |
| North East and Cumbria | 17.4% | (15.3%-19.7%) | 18.3% | (16.0%-20.8%) |
| Lancashire and South Cumbria | 17.1% | (14.2%-20.4%) | 15.8% | (13.0%-19.0%) |
| Greater Manchester | 18.7% | (16.4%-21.2%) | 17.3% | (15.1%-19.7%) |
| North Central and North East London | 27.8% | (24.9%-31.0%) | 24.2% | (21.4%-27.3%) |
| North West and South West London | 22.3% | (19.7%-25.2%) | 19.3% | (16.9%-22.1%) |
| p for difference | <0.001 |  | <0.001 |  |
| Covariate odds ratios |  |  |  |  |
| Per year increase in age |  |  | 0.95 | (0.95-0.96) |
| Female |  |  | 1.11 | (1.00-1.22) |
| Per quintile increase in deprivation quintile |  |  | 0.89 | (0.87-0.92) |

*Based on the income domain of the Index of Multiple Deprivation

**Supplementary Table 4:** **Age-standardised net one-year net survival by cohort and Cancer Alliance**

|  | 2010-2013 | | 2011-2014 | | 2012-2015 | | 2013-2016 | |
| --- | --- | --- | --- | --- | --- | --- | --- | --- |
|  | Net Surv. | 95% CI | Net Surv. | 95% CI | Net Surv. | 95% CI | Net Surv. | 95% CI |
| **All persons** |  |  |  |  |  |  |  |  |
| England Overall | 40.2% | (39.0%-41.4%) | 41.4% | (40.3%-42.6%) | 43.6% | (42.5%-44.7%) | 45.2% | (44.1%-46.3%) |
| West Yorkshire | 48.7% | (43.3%-53.9%) | 51.3% | (46.2%-56.3%) | 51.4% | (46.5%-56.1%) | 53.0% | (48.3%-57.5%) |
| Humber, Coast and Vale | 40.8% | (33.0%-48.5%) | 39.5% | (32.0%-46.8%) | 37.9% | (30.9%-44.8%) | 44.4% | (37.4%-51.3%) |
| Cheshire and Merseyside | 43.1% | (38.1%-48.1%) | 44.0% | (39.1%-48.7%) | 45.0% | (40.5%-49.4%) | 48.7% | (44.4%-53.0%) |
| South Yorkshire, Bassetlaw, North Derbyshire and Hardwick | 39.6% | (33.9%-45.3%) | 39.5% | (33.9%-45.0%) | 39.0% | (33.5%-44.5%) | 43.5% | (38.3%-48.6%) |
| West Midlands | 42.3% | (38.4%-46.2%) | 41.5% | (37.9%-45.1%) | 42.8% | (39.3%-46.2%) | 44.5% | (41.1%-47.8%) |
| East Midlands | 34.9% | (29.9%-40.0%) | 36.8% | (32.2%-41.5%) | 41.9% | (37.4%-46.4%) | 42.6% | (38.4%-46.8%) |
| East of England | 42.8% | (39.2%-46.4%) | 43.4% | (39.7%-46.9%) | 46.9% | (43.3%-50.4%) | 48.0% | (44.6%-51.4%) |
| South East London | 33.1% | (26.4%-40.0%) | 36.3% | (28.8%-43.8%) | 39.8% | (32.5%-47.0%) | 39.3% | (32.3%-46.3%) |
| Kent and Medway | 34.4% | (26.5%-42.4%) | 42.0% | (34.4%-49.4%) | 38.7% | (31.6%-45.7%) | 38.2% | (31.6%-44.7%) |
| Surrey and Sussex | 39.7% | (34.1%-45.2%) | 44.6% | (39.2%-49.9%) | 45.7% | (40.5%-50.8%) | 43.2% | (38.2%-48.1%) |
| Thames Valley | 44.7% | (37.2%-51.9%) | 43.7% | (36.8%-50.4%) | 43.3% | (37.2%-49.2%) | 44.4% | (38.5%-50.2%) |
| Peninsula | 45.5% | (38.4%-52.3%) | 45.2% | (38.7%-51.6%) | 52.3% | (46.1%-58.2%) | 51.1% | (45.2%-56.7%) |
| Somerset, Wiltshire, Avon and Gloucestershire | 37.8% | (32.0%-43.7%) | 39.6% | (34.2%-45.0%) | 44.4% | (39.3%-49.4%) | 46.0% | (41.1%-50.8%) |
| Wessex | 41.1% | (35.4%-46.7%) | 41.5% | (36.3%-46.6%) | 43.0% | (38.1%-47.8%) | 40.4% | (35.5%-45.1%) |
| North East and Cumbria | 43.8% | (39.4%-48.1%) | 43.4% | (39.1%-47.6%) | 43.9% | (39.8%-47.9%) | 45.5% | (41.3%-49.5%) |
| Lancashire and South Cumbria | 34.4% | (27.4%-41.5%) | 35.3% | (29.4%-41.2%) | 39.7% | (34.1%-45.2%) | 42.6% | (37.2%-48.0%) |
| Greater Manchester | 36.1% | (31.3%-41.0%) | 40.7% | (36.1%-45.3%) | 42.6% | (38.4%-46.8%) | 44.2% | (40.1%-48.3%) |
| North Central and North East London | 40.4% | (35.2%-45.6%) | 43.4% | (38.2%-48.5%) | 46.2% | (41.1%-51.2%) | 50.4% | (45.6%-55.0%) |
| North West and South West London | 38.8% | (33.4%-44.1%) | 39.9% | (34.6%-45.0%) | 41.8% | (36.7%-46.8%) | 44.1% | (39.3%-48.7%) |
|  |  |  |  |  |  |  |  |  |
| **Men** |  |  |  |  |  |  |  |  |
| England Overall | 40.0% | (38.6%-41.4%) | 41.3% | (39.9%-42.6%) | 43.6% | (42.3%-44.9%) | 45.3% | (44.1%-46.5%) |
| West Yorkshire | 49.6% | (43.3%-55.6%) | 48.9% | (43.0%-54.6%) | 49.1% | (43.5%-54.4%) | 51.3% | (46.0%-56.3%) |
| Humber, Coast and Vale | 30.4% | (21.9%-39.4%) | 35.6% | (27.2%-44.1%) | 36.5% | (28.7%-44.4%) | 45.5% | (37.7%-53.1%) |
| Cheshire and Merseyside | 44.1% | (38.1%-49.8%) | 45.3% | (39.6%-50.9%) | 46.6% | (41.2%-51.8%) | 51.2% | (46.1%-56.1%) |
| South Yorkshire, Bassetlaw, North Derbyshire and Hardwick | 36.0% | (29.5%-42.7%) | 39.5% | (33.0%-45.9%) | 38.6% | (32.4%-44.8%) | 44.2% | (38.3%-50.0%) |
| West Midlands | 43.6% | (39.1%-48.1%) | 42.1% | (37.8%-46.1%) | 42.5% | (38.5%-46.4%) | 44.5% | (40.6%-48.3%) |
| East Midlands | 35.3% | (29.6%-41.1%) | 39.1% | (33.7%-44.5%) | 44.3% | (39.1%-49.4%) | 44.6% | (39.9%-49.3%) |
| East of England | 43.2% | (39.0%-47.3%) | 43.8% | (39.6%-47.8%) | 48.0% | (43.9%-51.9%) | 48.4% | (44.4%-52.2%) |
| South East London | 33.0% | (25.6%-40.7%) | 37.1% | (28.8%-45.5%) | 40.5% | (32.4%-48.6%) | 38.0% | (30.1%-45.8%) |
| Kent and Medway | 30.3% | (21.8%-39.1%) | * | * | 37.6% | (29.4%-45.9%) | 37.0% | (29.5%-44.5%) |
| Surrey and Sussex | 39.7% | (33.3%-46.0%) | 44.7% | (38.6%-50.6%) | 45.5% | (39.7%-51.1%) | 43.5% | (37.9%-48.9%) |
| Thames Valley | 48.4% | (39.9%-56.4%) | 46.7% | (38.8%-54.3%) | 43.1% | (36.2%-49.8%) | 42.0% | (35.4%-48.4%) |
| Peninsula | 44.0% | (36%-51.8%) | 41.1% | (33.7%-48.4%) | 51.5% | (44.3%-58.3%) | 49.5% | (42.9%-55.8%) |
| Somerset, Wiltshire, Avon and Gloucestershire | * | * | 37.9% | (31.6%-44.2%) | 45.7% | (39.8%-51.3%) | 49.3% | (43.6%-54.7%) |
| Wessex | 39.3% | (33.1%-45.5%) | 42.3% | (36.5%-47.9%) | 43.2% | (37.7%-48.5%) | 41.4% | (35.9%-46.8%) |
| North East and Cumbria | 44.5% | (39.6%-49.3%) | 42.6% | (37.8%-47.3%) | 42.5% | (37.9%-47.1%) | 46.0% | (41.3%-50.5%) |
| Lancashire and South Cumbria | 32.8% | (25.5%-40.3%) | 33.0% | (26.6%-39.5%) | 38.2% | (32.0%-44.5%) | 39.6% | (33.4%-45.7%) |
| Greater Manchester | 36.3% | (30.8%-41.8%) | 41.3% | (36.0%-46.5%) | 42.7% | (37.9%-47.4%) | 44.6% | (40.0%-49.1%) |
| North Central and North East London | 39.7% | (33.9%-45.4%) | 44.3% | (38.5%-49.9%) | 46.2% | (40.5%-51.7%) | 51.3% | (45.8%-56.4%) |
| North West and South West London | 40.0% | (34.0%-46.0%) | 41.0% | (35.1%-46.8%) | 42.8% | (37.2%-48.4%) | 43.8% | (38.3%-49.1%) |
|  |  |  |  |  |  |  |  |  |
| **Women** |  |  |  |  |  |  |  |  |
| England Overall | 40.9% | (38.3%-43.5%) | 42.4% | (39.9%-44.9%) | 44.1% | (41.7%-46.4%) | 45.4% | (43.0%-47.7%) |
| West Yorkshire | 45.3% | (34.8%-55.3%) | 59.2% | (48.5%-68.5%) | 60.6% | (50.0%-69.6%) | 60.7% | (50.3%-69.7%) |
| Humber, Coast and Vale | 53.0% | (37.9%-66.2%) | 47.2% | (32.1%-61.1%) | * | * | * | * |
| Cheshire and Merseyside | 41.2% | (31.4%-50.8%) | 41.1% | (32.1%-49.9%) | 43.5% | (34.9%-51.8%) | 43.1% | (34.5%-51.5%) |
| South Yorkshire, Bassetlaw, North Derbyshire and Hardwick | 47.6% | (35.9%-58.4%) | 37.9% | (27.1%-48.6%) | 37.6% | (26.1%-49.1%) | 39.4% | (28.4%-50.2%) |
| West Midlands | 39.5% | (31.6%-47.3%) | 41.7% | (34.4%-48.8%) | 45.3% | (37.9%-52.4%) | 46.0% | (39.0%-52.6%) |
| East Midlands | 34.1% | (23.7%-44.7%) | 32.3% | (23.2%-41.8%) | 38.6% | (29.3%-47.8%) | 37.0% | (28.2%-45.8%) |
| East of England | 41.0% | (33.5%-48.4%) | 43.1% | (35.7%-50.3%) | 44.9% | (37.3%-52.2%) | 47.8% | (40.5%-54.8%) |
| South East London | * | * | ** | ** | ** | ** | 48.9% | (32.7%-63.4%) |
| Kent and Medway | ** | ** | 46.8% | (31.9%-60.5%) | 40.8% | (26.8%-54.3%) | 39.1% | (26.3%-51.7%) |
| Surrey and Sussex | 38.7% | (27.3%-49.9%) | 41.6% | (29.9%-53.0%) | 45.3% | (32.6%-57.2%) | * | * |
| Thames Valley | 26.8% | (13.2%-42.6%) | 20.0% | (9.4%-33.3%) | 41.2% | (28.1%-53.9%) | 50.9% | (37.2%-63.1%) |
| Peninsula | 53.2% | (37.5%-66.7%) | 57.6% | (43.5%-69.4%) | 53.5% | (40.9%-64.6%) | 61.1% | (47.6%-72.3%) |
| Somerset, Wiltshire, Avon and Gloucestershire | 31.9% | (21.2%-43.2%) | 36.7% | (25.9%-47.5%) | 41.7% | (31.4%-51.8%) | 37.7% | (27.7%-47.6%) |
| Wessex | 50.9% | (36.7%-63.5%) | 46.4% | (34.2%-57.9%) | 43.0% | (32.2%-53.4%) | 34.9% | (25.1%-44.8%) |
| North East and Cumbria | 41.2% | (31.8%-50.4%) | 46.7% | (36.7%-56.1%) | 46.1% | (37.3%-54.6%) | 46.7% | (37.6%-55.4%) |
| Lancashire and South Cumbria | ** | ** | 42.9% | (28.4%-56.7%) | 42.3% | (30.0%-54.2%) | 48.9% | (37.6%-59.3%) |
| Greater Manchester | 34.6% | (24.5%-45.0%) | 38.2% | (28.8%-47.6%) | 39.9% | (30.8%-48.9%) | 43.6% | (34.5%-52.4%) |
| North Central and North East London | 43.7% | (31.1%-55.6%) | 40.2% | (28.0%-52.2%) | 47.1% | (35.7%-57.8%) | 48.1% | (37.4%-58.0%) |
| North West and South West London | 35.1% | (23.7%-46.8%) | 37.4% | (26.1%-48.7%) | 41.5% | (30.3%-52.5%) | 47.6% | (37.4%-57.2%) |
| * Unable to calculate survival due to no deaths/data in one of the age bands | | | | | | | | |
| ** Results supressed due to high volatility or wide confidence intervals | | | | | | | | |

**Supplementary Table 5: Age-standardised net two-year net survival by cohort and Cancer Alliance**

|  | 2010-2013 | | 2011-2014 | | 2012-2015 | | 2013-2016 | | | |
| --- | --- | --- | --- | --- | --- | --- | --- | --- | --- | --- |
| **All Persons** |  |  |  |  |  |  |  |  | |  |
| England overall | 27.8% | (26.5%-29.1%) | 29.4% | (28.2%-30.6%) | 31.4% | (30.2%-32.5%) | 32.8% | (31.7%-34.0%) | |  |
| West Yorkshire | 34.8% | (29.1%-40.6%) | 37.2% | (31.7%-42.7%) | 40.0% | (34.8%-45.1%) | 39.8% | (34.9%-44.7%) | |  |
| Humber, Coast and Vale | 28.3% | (20.8%-36.3%) | 30.0% | (22.6%-37.7%) | 25.7% | (19.0%-32.9%) | 31.8% | (24.7%-39.2%) | |  |
| Cheshire and Merseyside | 31.7% | (26.5%-37.0%) | 32.3% | (27.4%-37.5%) | 32.6% | (27.8%-37.4%) | 36.4% | (31.8%-40.9%) | |  |
| South Yorkshire, Bassetlaw, North Derbyshire and Hardwick | 26.3% | (20.8%-32.1%) | 27.1% | (21.7%-32.7%) | 25.7% | (20.4%-31.3%) | 30.2% | (25.0%-35.6%) | |  |
| West Midlands | 29.9% | (25.8%-34.0%) | 27.4% | (23.8%-31.0%) | 29.1% | (25.6%-32.7%) | 34.0% | (30.6%-37.5%) | |  |
| East Midlands | 25.2% | (20.2%-30.4%) | 28.0% | (23.3%-32.9%) | 31.3% | (26.7%-36.1%) | 28.7% | (24.5%-32.9%) | |  |
| East of England | 27.8% | (24.2%-31.5%) | 31.2% | (27.6%-34.9%) | 34.5% | (30.9%-38.2%) | 34.9% | (31.3%-38.5%) | |  |
| South East London | 25.0% | (18.5%-32.0%) | 28.9% | (21.5%-36.8%) | 34.0% | (26.5%-41.7%) | 32.1% | (25.1%-39.4%) | |  |
| Kent and Medway | 24.6% | (17.1%-32.8%) | 30.3% | (22.8%-38.1%) | 24.5% | (17.6%-31.9%) | 26.2% | (19.9%-32.9%) | |  |
| Surrey and Sussex | 24.5% | (19.2%-30.2%) | 34.2% | (28.7%-39.9%) | 36.9% | (31.6%-42.3%) | 29.4% | (24.4%-34.6%) | |  |
| Thames Valley | 32.9% | (25.4%-40.7%) | 29.0% | (22.1%-36.3%) | 31.3% | (25.2%-37.6%) | 31.0% | (25.1%-37.1%) | |  |
| Peninsula | 33.3% | (25.9%-40.8%) | 35.4% | (28.6%-42.3%) | 39.7% | (33.2%-46.2%) | 39.8% | (33.8%-45.8%) | |  |
| Somerset, Wiltshire, Avon and Gloucestershire | 29.0% | (23.0%-35.2%) | 30.6% | (25.1%-36.3%) | 32.4% | (27.1%-37.9%) | 34.4% | (29.3%-39.5%) | |  |
| Wessex | 30.0% | (24.2%-36.0%) | 31.0% | (25.8%-36.4%) | 32.6% | (27.7%-37.6%) | 27.4% | (22.7%-32.2%) | |  |
| North East and Cumbria | 30.5% | (26.1%-35.0%) | 31.7% | (27.4%-36.2%) | 29.8% | (25.7%-34.0%) | 31.3% | (27.2%-35.5%) | |  |
| Lancashire and South Cumbria | 21.2% | (14.6%-28.6%) | 22.2% | (16.7%-28.2%) | 24.2% | (18.9%-29.9%) | 28.0% | (22.7%-33.5%) | |  |
| Greater Manchester | 22.7% | (18.1%-27.6%) | 28.4% | (23.7%-33.2%) | 28.8% | (24.6%-33.2%) | 31.6% | (27.5%-35.8%) | |  |
| North Central and North East London | 27.2% | (22.0%-32.5%) | 28.5% | (23.3%-34.0%) | 33.5% | (28.3%-38.9%) | 38.0% | (32.9%-43.1%) | |  |
| North West and South West London | 27.8% | (22.5%-33.3%) | 27.7% | (22.6%-33.1%) | 29.5% | (24.5%-34.6%) | 31.6% | (26.8%-36.6%) | |  |
|  |  |  |  |  |  |  |  |  | |  |
| **Male** |  |  |  |  |  |  |  |  | |  |
| England overall | 27.5% | (26.0%-28.9%) | 28.9% | (27.5%-30.2%) | 31.3% | (29.9%-32.6%) | 32.6% | (31.4%-33.9%) | |  |
| West Yorkshire | 33.0% | (26.5%-39.7%) | 33.0% | (27.0%-39.2%) | 37.2% | (31.4%-43.0%) | 38.0% | (32.5%-43.6%) | |  |
| Humber, Coast and Vale | 23.0% | (14.8%-32.4%) | 27.2% | (18.7%-36.5%) | 23.5% | (16.2%-31.6%) | 32.7% | (24.7%-40.9%) | |  |
| Cheshire and Merseyside | 32.5% | (26.5%-38.7%) | 33.3% | (27.3%-39.4%) | 33.0% | (27.4%-38.8%) | 37.9% | (32.6%-43.2%) | |  |
| South Yorkshire, Bassetlaw, North Derbyshire and Hardwick | 21.8% | (16.0%-28.4%) | 25.5% | (19.3%-32.2%) | 25.4% | (19.5%-31.8%) | 30.8% | (24.9%-36.9%) | |  |
| West Midlands | 30.1% | (25.5%-34.9%) | 26.6% | (22.6%-30.9%) | 27.9% | (24.0%-32.0%) | 32.4% | (28.4%-36.4%) | |  |
| East Midlands | 25.0% | (19.4%-31.1%) | 28.9% | (23.4%-34.7%) | 32.1% | (26.8%-37.6%) | 29.6% | (25.0%-34.4%) | |  |
| East of England | 28.5% | (24.4%-32.7%) | 32.1% | (27.9%-36.3%) | 35.6% | (31.3%-39.8%) | 35.5% | (31.4%-39.7%) | |  |
| South East London | 23.9% | (16.8%-31.8%) | 28.1% | (19.8%-37.0%) | 33.0% | (24.7%-41.7%) | 31.1% | (23.3%-39.3%) | |  |
| Kent and Medway | 22.0% | (14.0%-31.2%) | * | * | 21.6% | (14.2%-30.3%) | 22.9% | (16.0%-30.6%) | |  |
| Surrey and Sussex | 24.6% | (18.5%-31.2%) | 36.6% | (30.2%-43.0%) | 37.6% | (31.7%-43.5%) | 29.5% | (24.0%-35.2%) | |  |
| Thames Valley | 33.2% | (24.5%-42.3%) | 30.8% | (22.8%-39.1%) | 32.8% | (25.9%-39.9%) | 29.4% | (23.0%-36.2%) | |  |
| Peninsula | 32.0% | (23.8%-40.4%) | 34.0% | (26.4%-41.8%) | 44.4% | (36.8%-51.7%) | 42.7% | (35.9%-49.3%) | |  |
| Somerset, Wiltshire, Avon and Gloucestershire | * | * | 29.1% | (22.7%-35.7%) | 33.6% | (27.4%-39.9%) | 37.2% | (31.3%-43.1%) | |  |
| Wessex | 29.2% | (22.9%-35.8%) | 31.7% | (26.0%-37.7%) | 32.0% | (26.5%-37.7%) | 27.3% | (21.9%-33.0%) | |  |
| North East and Cumbria | 31.2% | (26.3%-36.3%) | 31.3% | (26.6%-36.1%) | 29.7% | (25.1%-34.5%) | 30.7% | (26.0%-35.6%) | |  |
| Lancashire and South Cumbria | 19.8% | (13.0%-27.7%) | 19.5% | (13.9%-26.0%) | 22.6% | (16.9%-28.9%) | 26.3% | (20.4%-32.5%) | |  |
| Greater Manchester | 21.6% | (16.5%-27.1%) | 28.4% | (23.1%-33.9%) | 29.0% | (24.2%-33.9%) | 31.4% | (26.8%-36.1%) | |  |
| North Central and North East London | 26.4% | (20.9%-32.3%) | 28.3% | (22.5%-34.4%) | 32.8% | (27.0%-38.9%) | 37.5% | (31.7%-43.2%) | |  |
| North West and South West London | 29.0% | (23.0%-35.3%) | 29.2% | (23.3%-35.3%) | 30.7% | (25.1%-36.5%) | 30.5% | (25.2%-36.0%) | |  |
|  |  |  |  |  |  |  |  |  | |  |
| **Female** |  |  |  |  |  |  |  |  | |  |
| England overall | 29.0% | (26.4%-31.7%) | 31.4% | (28.9%-34.0%) | 32.0% | (29.6%-34.5%) | 34.0% | (31.6%-36.4%) | |  |
| West Yorkshire | 40.5% | (29.6%-51.3%) | 51.6% | (39.9%-62.3%) | 50.1% | (38.9%-60.5%) | 47.1% | (36.2%-57.4%) | |  |
| Humber, Coast and Vale | 35.2% | (20.7%-50.3%) | 36.1% | (21.8%-50.7%) | * | * | * | * | |  |
| Cheshire and Merseyside | 30.3% | (20.6%-40.7%) | 30.8% | (22.0%-40.2%) | 32.7% | (24.0%-41.8%) | 31.8% | (23.5%-40.5%) | |  |
| South Yorkshire, Bassetlaw, North Derbyshire and Hardwick | 35.0% | (23.6%-46.6%) | 29.7% | (19.6%-40.7%) | 25.4% | (14.9%-37.4%) | 26.1% | (16.1%-37.4%) | |  |
| West Midlands | 29.5% | (21.5%-37.9%) | 31.0% | (23.8%-38.6%) | 35.9% | (28.3%-43.5%) | 40.8% | (33.8%-47.8%) | |  |
| East Midlands | 24.5% | (14.7%-35.7%) | 26.8% | (18.0%-36.4%) | 32.4% | (22.8%-42.5%) | 26.8% | (18.1%-36.2%) | |  |
| East of England | 23.3% | (16.5%-30.9%) | 27.9% | (20.9%-35.4%) | 31.7% | (24.4%-39.4%) | 33.3% | (26.0%-40.8%) | |  |
| South East London | * | * | ** | ** | ** | ** | ** | ** | |  |
| Kent and Medway | ** | ** | 33.9% | (19.4%-49.2%) | 31.2% | (17.3%-46.3%) | 34.1% | (21.4%-47.3%) | |  |
| Surrey and Sussex | 24.9% | (14.4%-37.0%) | 25.8% | (15.4%-37.7%) | 31.8% | (20.0%-44.4%) | * | * | |  |
| Thames Valley | 27.3% | (13.5%-43.3%) | 13.9% | (5.0%-27.4%) | 27.4% | (14.4%-42.2%) | 36.1% | (22.7%-49.7%) | |  |
| Peninsula | ** | ** | 37.4% | (23.5%-51.4%) | 22.9% | (13.2%-34.4%) | 34.9% | (21.9%-48.3%) | |  |
| Somerset, Wiltshire, Avon and Gloucestershire | 20.8% | (11.3%-32.3%) | 27.0% | (16.6%-38.5%) | 30.2% | (19.9%-41.2%) | 28.7% | (18.8%-39.5%) | |  |
| Wessex | 35.8% | (21.7%-50.2%) | 36.2% | (24.0%-48.7%) | 34.4% | (23.7%-45.5%) | 22.9% | (14.4%-32.7%) | |  |
| North East and Cumbria | 27.4% | (18.5%-37.1%) | 33.8% | (23.5%-44.4%) | 28.2% | (19.7%-37.3%) | 32.2% | (23.6%-41.2%) | |  |
| Lancashire and South Cumbria | ** | ** | 31.3% | (16.9%-47.1%) | 26.1% | (14.5%-39.3%) | 29.7% | (19.0%-41.4%) | |  |
| Greater Manchester | 28.3% | (18.0%-39.6%) | 29.3% | (20.1%-39.2%) | 24.4% | (16.0%-34.0%) | 29.7% | (20.7%-39.4%) | |  |
| North Central and North East London | 31.0% | (18.8%-44.2%) | 32.6% | (20.6%-45.3%) | 38.1% | (26.3%-49.9%) | 42.2% | (31.2%-53.0%) | |  |
| North West and South West London | 21.9% | (11.8%-34.2%) | 23.4% | (13.2%-35.4%) | 28.3% | (17.6%-39.9%) | 39.7% | (28.8%-50.6%) | |  |
| * Unable to calculate survival due to no deaths/data in one of the age bands | | | | | | | | |  |  |
| ** Results supressed due to high volatility or wide confidence intervals | | | | | | | | |  |  |

**Supplementary Table 6: Correlation of Study Metric between Cancer Alliances**

|  | % in the most deprived† | Incidence ASR 2013 Men | Incidence ASR 2013 Women | % Emergency Presentations (adjusted‡) | % Curative Treatment (adjusted‡) | One-year Net Survival | Two-year Net Survival |
| --- | --- | --- | --- | --- | --- | --- | --- |
| % in the most deprived quintile | 1 |  |  |  |  |  |  |
| Incidence ASR 2013 Men | 0.740* | 1 |  |  |  |  |  |
| Incidence ASR 2013 Women | 0.704* | 0.751* | 1 |  |  |  |  |
| % Emergency Presentations (adjusted) | 0.007 | -0.051 | -0.040 | 1 |  |  |  |
| % Curative Treatment (adjusted) | 0.246 | 0.241 | 0.246 | -0.353 | 1 |  |  |
| One-year Net Survival | 0.217 | 0.314 | 0.201 | -0.531* | 0.795* | 1 |  |
| Two-year Net Survival | 0.262 | 0.231 | 0.212 | -0.527* | 0.766* | 0.914* | 1 |

*p values in less than 0.05

†proportion of the whole population in the most deprived quintile

‡adjusted for age, sex and IMD
